# Estimation of Density Distribution in a Rigid PU Foam Block Manufactured in a Sealed Mold

**DOI:** 10.3390/polym18060733

**Published:** 2026-03-17

**Authors:** Ilze Beverte, Ugis Cabulis, Jānis Andersons

**Affiliations:** 1 Institute for Mechanics of Materials, University of Latvia, 3 Jelgavas St., LV-1004 Riga, Latvia; a09660418@gmail.com (I.B.); janis.andersons@pmi.lv (J.A.); 2 Latvian State Institute of Wood Chemistry, Dzērbenes Street 27, LV-1006 Riga, Latvia

**Keywords:** polyurethane foams, sealed mold, graded density, distribution, symmetry, approximation, polynomials, ellipse

## Abstract

Rigid polyurethane foams are often manufactured in sealed molds, so knowledge of the density distribution in the molded blocks is essential. A study was conducted with the aim to estimate density distribution within a rigid polyurethane foam block (average core density of ≈96 kg/m^3^) manufactured in a rectangular sealed mold. The density of 150 rectangular samples was determined experimentally. Characteristic locations of the foams’ columns in the block were outlined, having similar foaming conditions. Averaged density in the characteristic columns was calculated for each characteristic location. A mathematical model was developed based on density data of characteristic columns, approximated with second- and third-degree polynomials. Density distribution was calculated, and corresponding color charts with density zones and equidensity lines were constructed for six horizontal and two vertical sections of the block. It was found that the common center of the elliptical equidensity lines is located asymmetrically, ≈17 mm above the geometric center of the untrimmed block. Density gradients were calculated in directions parallel and perpendicular to the foams’ rise direction. The developed mathematical model allowed us to estimate density distribution within the rigid polyurethane foam block manufactured in a rectangular sealed mold.

## 1. Introduction

Rigid polyurethane (PU) foams are polymer–gas composites with outstanding thermal insulation properties and acceptable load bearing capacity, which are applied in various engineering solutions [1,2]. The foams are used for both heat- and cold-saving insulation in stores, warehouse buildings, and constructions; as thermal insulation of mortars for masonry; in the construction sector in sandwich structures and panels; in the transport sector for cooling vans and vehicle insulation; as cryogenic insulation in aircrafts and spacecrafts; in radomes for providing a radio-frequency transparent protective layer; etc. [3].

The two main technologies for manufacturing blocks of rigid PU foams are (1) free-rise in an open mold and (2) foaming in a sealed mold [4,5,6,7,8]. For rigid free-rise PU foams, density distribution and density gradients are studied at different sizes and shapes of molds, as well as processing temperatures [9,10,11,12,13,14].

Various modifications of sealed molds have been used to obtain rigid PU foams in lab conditions. In [15], a mixed liquid composition of PU foams was poured into cylindrical molds at room temperature to produce PU foams of density 100–400 kg/m^3^. The molds were then closed, and the foam was allowed to expand to fill the closed molds at a packed density of approximately 1.75 times the expected free-rise density. In [16], the molds used for foaming PU foams CRETE consisted of two steel plates, perforated with small holes to allow gas and foam escape, with steel cylinders of various heights and diameters between the plates. Rigid PU foams from petrochemical and renewable components were made in a sealed metallic mold measuring 140 × 140 × 50 mm [17]. In [18], the liquid reacting mixture of PU foams, infused with nanoparticles, with an expected density of 240 kg/m^3^, was poured into a preheated aluminum mold, made as two aluminum plates on both sides of an aluminum frame. Closed plastic containers were used for the manufacturing of PU/clay nanoparticle-filled foams in [19]. The density distribution in the molded blocks was not considered in the abovementioned studies [15,16,17,18,19].

Sealed molds are widely used in studies of PU foams filled with various nanoparticles (spherical TiO_2_, platelet–nanoclays, rod-shaped carbon nanofibers, etc.) [20,21,22]. When neat and nanoparticle-filled PU foams are produced in an open mold, the degree of monotropy of filled PU foams differs from that of neat foams, since the foams rise to different heights. To study the impact of organoclays Cloisite 15A and Cloisite 30B on the mechanical properties of rigid closed-cell PU foams, the foams of targeted apparent density 200 kg/m^3^ were made in a sealed metallic mold [20]. As identified by SEM microscopy, the geometrical isotropy of cells was virtually unaffected by the filler. The slight deviations from isotropic material were explained by the difference in apparent densities of the samples, since foams near the mold walls have an increased density. Light microscopy of rigid, neat nanoclay-filled PU foams (density ≈ 250 kg/m^3^) manufactured in a sealed steel mold showed local isotropy of the foams’ structure [21].

In a study on sandwich panel manufacturing, a sealed mold and overpressure was used to obtain an isotropic PU foam core [22]. The closeness of the PU foams’ structure to the isotropic one depended on how high the overpressure was. In sandwich panels manufactured at a lower overpressure, an anisotropic structure was observed [23]. In [24], rigid PU foam blocks shaped as a truncated pyramid were produced in a sealed steel mold for study of nanoclays’ impact. At a proper choice of technological parameters, foaming in a sealed mold allowed for the manufacturing of locally nearly isotropic PU foams, thus reducing the influence of anisotropy variations. Locations with similar foaming conditions and similar expected density were identified in [25]. Density distribution was estimated based on data of compression and tension samples, but the number of samples was too small to draw broader conclusions.

Research results on density distribution in blocks manufactured in lab conditions, in sealed molds, are fragmented and insufficient and do not allow the density distribution to be assessed throughout the volume of a block. Therefore, the aim of this study is to investigate experimentally and model mathematically the density distribution in a rectangular block of rigid PU foams manufactured in a sealed plywood mold, without preheating. Characteristic locations of the foams’ columns in the block were outlined, having similar foaming conditions. A mathematical model was elaborated, based on density data of characteristic columns, approximated with second- and third-degree polynomials. Novelty lies in constructing density charts for the foam block based on the averaged experimental data of characteristic columns and introducing the concept of equidensity lines. This allowed density zones to be visualized and their shape parameters to be estimated. It was found that the foaming center is located asymmetrically, above the geometric center of untrimmed block.

## 2. Materials and Methods

### 2.1. Manufacturing of the PU Foam Block

**Figure 1:**
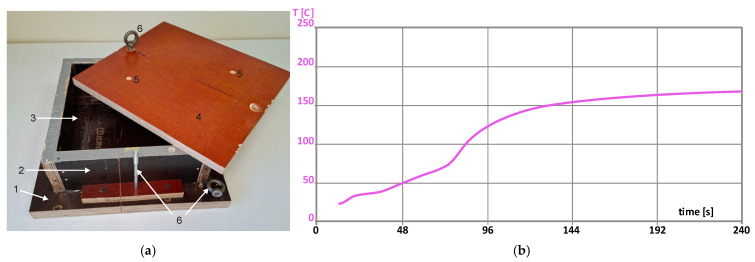
(**a**) The sealable plywood mold: (1) base, (2) body with (3) working volume, (4) lid with (5) two gas release holes, and (6) a screw and fixing nuts. (**b**) The temperature profile of the foaming.

To manufacture a block of conventional rigid closed-cell PU foams, the following raw materials and their ratio in parts by weight per 100 parts of polyol (pbw) were used (Table 1). Foaming was performed in a detachable, reusable sealed plywood mold at approximately double overpressure. The value of overpressure in the sealed mold was calculated as p_ov_ = ρ_sm_/ρ_0_, where ρ_sm_ is the apparent overall density of a block manufactured in a sealed mold, and ρ_0_ is the same for a block manufactured in an open mold (ISO 845:2006). Density of free-rise PU foams of the given formulation was determined, manufacturing foams in an open mold of the same transversal dimensions as those of the sealable mold [24]. The dimensions of the working volume of the mold were 250 mm × 250 mm × 130 mm (Figure 1a). Since the isocyanate index was II = 130, the amount of the necessary MDI was adjusted to 168 pbw to compensate for the water.

To prepare the polyol composition, all components were weighted on a lab scale and mixed with a mechanical stirrer. Then, the polyol system was conditioned at room temperature for 24 h in a sealed container to remove air. To manufacture the foam block, the polyol system and MDI were mixed with the mechanical stirrer (2400 rpm ± 20 rpm) for 10 s, and the mixture was poured into the mold. The mold was at room temperature, without preheating. The technology relays on the low thermal conductivity of plywood 0.10 W/(m·K) < λ < 0.14 W/(m·K). Other materials, commonly used for molds, have considerably higher values of λ: (a) common alloys of aluminum, 227–237 W/(m·K); (b) carbon steels, 45–60 W/(m·K); mild steels, 46–50 W/(m·K); stainless steels, 15–25 W/(m·K); and (c) plastics, 0.1–0.5 W/(m·K). Then, the mold was sealed, and the composition was allowed to expand to a density app. twice the expected free-rise density of ≈50 kg/m^3^. The polymerization reaction took place at room temperature and was completed in app. 30–45 min. Foaming parameters were as follows: stirring, 15 s; start time, 25 s; and foaming end time, 180 s. The temperature profile of foaming is given in Figure 1b. A thermocouple connected with Universal Foam Qualification System FOAMAT (Format Messtechnik GmbH, Karlsruhe, Germany) for measuring of temperature inside the PU foam block was used. The thin thermocouple is suited for measuring the temperature inside the foam because it has a low heat capacity and has virtually no effect on the foams’ formation.

The dimensions of the manufactured block with skins were ≈250 mm × 250 mm × 130 mm. After cutting off a layer of ≈10 mm thickness from the entire outer surface of the block and grinding the surfaces, a parallelepiped with dimensions of 230 mm × 230 mm × 100 mm was obtained. The average density of the trimmed block was determined as 96.2 kg/m^3^.

### 2.2. Density of Samples

**Figure 2:**
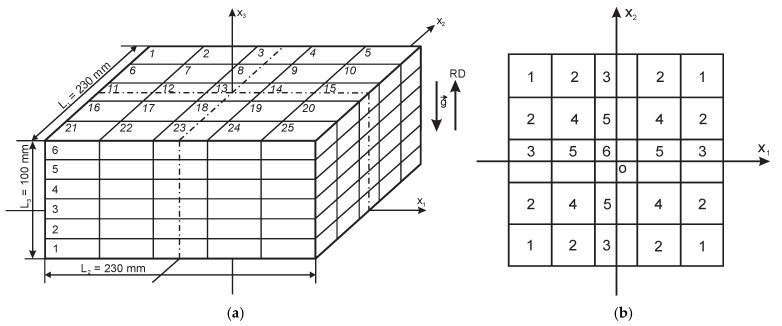
(**a**) Division of the PU foam block into 25 columns and six horizontal layers. (**b**) Six characteristic locations of columns in the block ( $\vec{g}$ —Earth’s gravity vector; and RD—rise direction of foams).

The trimmed block of PU foams was divided into 25 vertical columns and 6 horizontal layers (Figure 2a). Each column comprised six samples, numbered from the bottom to the top of a column, parallel to the rise direction, ox_3_, yielding N = 150 rectangular, parallelepiped-shaped samples. The dimensions of a sample in an uncut block were 46 mm × 46 mm × 17 mm. Cutting was made with a band saw, with cutting and grinding allowances of ≈2.0 mm. The final dimensions of the samples were ≈44 mm × 44 mm × 15 mm.

The density of samples was determined according to EN ISO 1923:1995 and ISO 845:2006 [26,27], at ambient temperature of 20 °C ≤ T ≤ 22 °C and relative humidity of 49% ≤ RH ≤ 53%; no conditioning was made. Temperature and relative humidity were measured with a thermo-hygrometer Testo 608-H1 (Testo SE & Co. KGaA, Lenzkirch, Germany); the expanded uncertainties were U95% = ±0.22°C and U95% = ±2.2%. The mass was measured with laboratory balance PS R2 (Radwag—Headquarters, Radom, Poland), max weight of 210 g, readability of 0.01 g, and accuracy of 0.02 g. The linear dimensions were measured with electronic digital caliper Mitutoyo Vernier 500-196-230 0–300 mm/12” (Mitutoyo Europe GmbH, Neuss, Germany), range of 0–300 mm, resolution of 0.01 mm, and accuracy of 0.02 mm + 0.00005 L mm (L—length of an object). Three measurements were made for each data point of mass and dimensions; then, the density of the samples was calculated as

(1) $$\rho_{n}=\frac{m_{nav}}{l_{1nav}l_{2nav}l_{3nav}},\;m_{nav}=\frac{1}{3}\sum_{i=1}^{3}m_{ni},\;\;l_{1nav}=\frac{1}{3}\sum_{i=1}^{3}l_{1ni},\;l_{2nav}=\frac{1}{3}\sum_{i=1}^{3}l_{2ni},\;l_{3nav}=\frac{1}{3}\sum_{i=1}^{3}l_{3ni}.$$

where l_1n_, l_2n_, and l_3n_ are the dimensions and m_n_ is the mass of the n-th sample, and n = 1, 2, …, 150 (Table S1 of Supplementary Materials). 

Taking into account the random (Type A) and the systematic (Type B) components of uncertainty, the expanded uncertainty of density measurements of 87 kg/m^3^ ≤ ρ ≤ 109 kg/m^3^ was estimated. Density of a sample (ρ) was treated as a function of four uncorrelated random variables: mass, m; and linear dimensions, l_1_, l_2_, and l_3_, of a rectangular sample. It was assumed that the values of density function follow a normal distribution. The density function was considered to be sufficiently smooth, and at small uncertainties, its variance was calculated by the delta method. The uncertainty components introduced by accuracy and precision of the laboratory balance and the electronic caliper were taken into account. The effective degrees of freedom, ν_eff_, of the combined standard uncertainty, u_c_(ρ), associated with the output estimate of ρ were estimated from the Welch–Satterthwaite formula. With ν_eff_ known, the coverage factor, k, was determined from numerical tables, based on t-distribution evaluated for a coverage probability of 95.45%. Then, the expanded uncertainty was estimated: U(ρ)_95%_ = ku_c_(ρ) < 1.7 kg/m^3^ [28,29,30].

### 2.3. Microscopy

Two microscopy samples, size 5 mm × 5 mm × 5 mm, were cut from layers No. 2 and 5 each. The coordinates of the geometric centers of the samples in the block were calculated (Table 2 and Page S2 in Supplementary Materials), taking into account that the layer height in an uncut block equals 17 mm (Figure 2a). Density of the samples was determined according to ISO 845:2006.

Thin slices (thickness ≈ 0.10 mm) were cut as narrow-angled wedges with a razor blade from three mutually perpendicular faces of each sample. Each sample yielded three slices: parallel to the (1) x_1_ox_2_, (2) x_1_ox_3_, and (3) x_2_ox_3_ planes. The slices were fixed on a glass slide, and rise direction, ox_3_, was marked. A light microscope Diamond MCXMP500, MICROS Produktions & Handels GmbH, St. Veit an der Glan, Austria, with processing software and calibration reticle was used to study the structure of foams at magnification of 10×. Diameters d_1_ and d_2_ of 35 cells’ faces (i.e., projections of the faces on photo plane) were measured from the onscreen images of the x_1_ox_2_ plane slices, d_1_ and d_3_ from the x_1_ox_2_ plane slices, and d_2_ and d_3_ from the x_2_ox_3_ plane slices, where d_i_ is the biggest dimension of a cell’s face along direction ox_i_, and i = 1, 2, or 3. Anisotropy degree AD was calculated for each face in dependence of the plane of the slice [7,8,20,21]:

AD_12_ = d_2_/d_1_, AD_13_ = d_3_/d_1_, or AD_23_ = d_3_/d_2_. (2)

Then, the average anisotropy degree was calculated for foams of each slice, which was parallel to the x_1_ox_2_, x_1_ox_3_, or x_2_ox_3_ plane:

(3) $${AD}_{12av}=\frac{1}{35}\sum_{i=1}^{35}{AD}_{12i},\;{AD}_{13av}=\frac{1}{35}\sum_{i=1}^{35}{AD}_{13i},\;\mathrm{or}\;{AD}_{23av}=\frac{1}{35}\sum_{i=1}^{35}{AD}_{23i}.$$

The load-bearing elements—the polymeric struts, frame faces of cells [6,7,8], anisotropy degree characteriszs spatial orientation of the struts.

### 2.4. Density in Characteristic Columns

Characteristic locations of columns in the block were outlined, having similar foaming conditions (theoretically identical) [24,25]. At the given division of the block into 25 columns, 6 kinds of locations (j = 6) were identified (Figure 2b). In a sealed mold, the columns have contact with the adjacent columns and the mold, but not with the surrounding environment. The number of contact surfaces for each column at a certain characteristic location is given in Table 3.

Theoretically, under (1) ideal foaming conditions of the block and (2) accurate measurements of dimensions and mass, the density of samples in columns from locations of the same kind should be equal. In practice, the raw density data showed deviations from equality (Table S1 of Supplementary Materials). Therefore, the density was averaged across same-name samples from all columns in locations of the same kind, yielding a characteristic column of a certain kind of location. The average density of the i-th sample in a characteristic column at the location of the j-th kind was calculated:

(4) $$\rho_{avj}^{i}=\frac{1}{{NC}_{j}}\sum_{nc=1}^{{NC}_{j}}\rho_{nc}^{i},$$

where i = 1, 2, …, 6; j = 1, 2, …, 6; and NC_j_—number of columns in the location of j-th kind (Table 4). It can be seen that |σ| ≤ 1.3 kg/m^3^ and v ≤ 1.2% for all kinds of locations, meaning that the foaming conditions have been quite similar in locations of the same kind.

**Figure 3:**
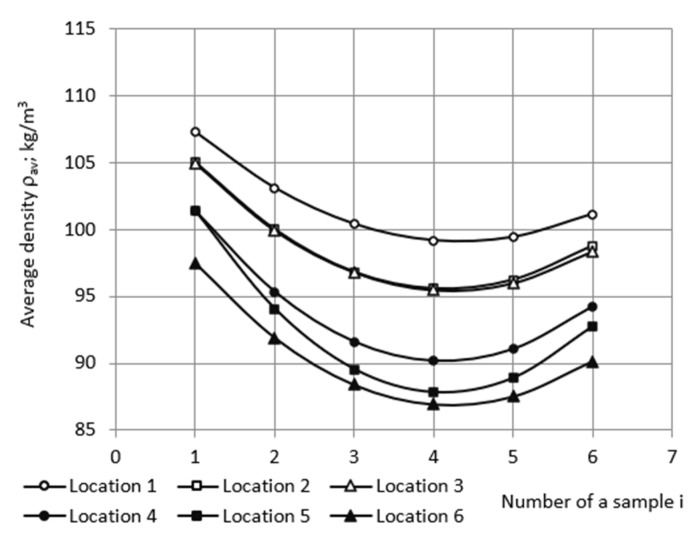
The fitted average density depending on the ordinal number of samples in the characteristic columns.

To smooth out deviations of the average dens, ity from the general trend within a characteristic column, trendlines were determined for the average density depending on the ordinal number of a sample in the characteristic column. Equations were found as second-degree polynomials (Parabolas):

ρ_av_ = A_1_i^2^ + B_1_i + C_1_; (5)

where A_1_, B_1_, and C_1_ are coefficients and i is the ordinal number of a sample in the j-th characteristic column; i = 1, 2, …, 6. From here on, all approximations were made using MS EXCEL v16.0 software (Microsoft Corporation; Redmond, WA, USA). The fitted average density of samples was calculated from trendline Equation (5) for the characteristic columns (Table 5 and Figure 3). The coefficients of determination 0.91 ≤ R^2^ ≤ 0.97 confirmed the need to smooth deviations of average density from the general trend within the characteristic column.

Using the data from Table 3 and following the scheme in Figure 2b, a basic matrix of fitted density data for the entire block was obtained (Table S2 of the Supplementary Materials).

### 2.5. Density in Horizontal Layers

The dimensions of the horizontal layers No. 1–6 in an uncut block are 230 mm × 230 mm × 17 mm. In order to estimate density distribution in the horizontal layers, which are parallel to the plane x_1_ox_2_ (Figure 2a), the relative coordinates ξ_1_, ξ_2_, and ξ_3_ were defined as follows:

ξ_1_ = x_1_/(L_1_/2), ξ_2_ = x_2_/(L_2_/2), and ξ_3_ = x_3_/L_3_, (6)

where L_1_ = L_2_ = 230 mm and L_3_ = 100 mm are the dimensions of the trimmed block. Since the dimensions of a sample in an uncut block equal 46 mm × 46 mm × 17 mm, the absolute and relative coordinates of the centers of the 150 uncut samples can be calculated (Tables S3–S5 of the Supplementary Materials).

A basic density matrix [5 × 5] was formed for each horizontal layer, Nos. 1, 2, …, 6, based on the fitted average density data of the characteristic columns (Table 3). The data of all samples for No. 1 were grouped into the basic density matrix of layer No. 1, the data of all samples No. 2 into the basic density matrix of layer No. 2, etc. Each basic density matrix comprises 25 data points (Tables S6–S11 of the Supplementary Materials).

First, each row of the basic density matrix of a layer was approximated depending on the relative coordinate, ξ_1_. Data had the best correlation with second-degree polynomials:

ρ = A_2_(ξ_1_)^2^ + B_2_ξ_1_ + C_2_; (7)

where A_2_, B_2_, and C_2_ are coefficients. The coefficient of determination was R^2^ > 96% for all layers, while for polynomials of other degrees and for other functions (e.g., the exponent), the value of R^2^ was considerably lower. That determined the final choice of model functions as second-degree polynomials (considerations in other cases of approximations were similar). Segments of other functions (normal and lognormal probability density functions and cumulative density functions) were tested in approximation as well. They fitted some data cases better than the polynomials (7) but gave insufficiently consistent overall results and were thus discarded.

Due to the fourth-order rotational symmetry, C_4_, of the foam block around the ox_3_ axis [24,25,31,32], only that part, 115 mm × 115 mm × 17 mm, of each layer is considered further, which corresponds to the first quadrant of the x_1_ox_2_ plane: 0.0 ≤ x_1_, x_2_ ≤ L_1_/2 = L_2_/2 = 115 mm (Figure 2a). That allows us to reduce the volume of numerical calculations. Then, for a more detailed analysis, the number of data points was increased to 20 + 1 = 21 (including point zero) along the ox_1_ axis. The corresponding step equals Δx_1_ = L_1_/2: 20 = 115 mm: 20 = 5.8 mm. Then, Δξ_1_ = Δx_1_/(L_1_/2) = 0.050 = 5%. Using Equation (7), the density in each of the five rows of the basic matrix was calculated in 21 data points.

The number of data points was increased to 20 + 1 = 21 along the ox_2_ axis as well. The step Δx_2_ = L_2_/2: 20 = 115 mm: 20 = 5.8 mm. Then, Δξ_2_ = Δx_2_/(L_2_/2) = 0.05 = 5%, and Δξ_2_ = Δξ_1_. Then, the elementary material unit in the x_1_ox_2_ plane is approximately a square Δx_1_ × Δx_2_. Based on the calculated density values in the five rows, approximating equations were determined for each column of the expanded basic matrix. Data had the best correlation with second-degree polynomials:

ρ = A_3_(ξ_2_)^2^ + B_3_ξ_2_ + C_3_; (8)

where A_3_, B_3_, and C_3_ are coefficients. Using the Equation (8), the density in each of the 21 columns of the expanded density matrix was calculated at 21 data points. The expanded density matrices [21 × 21] of horizontal layers are given in Tables S12–S17 of the Supplementary Materials, each comprising 21 × 21 = 441 elements.

### 2.6. Density in Vertical Sections

#### 2.6.1. Vertical Section Perpendicular to the Side of Block

**Figure 4:**
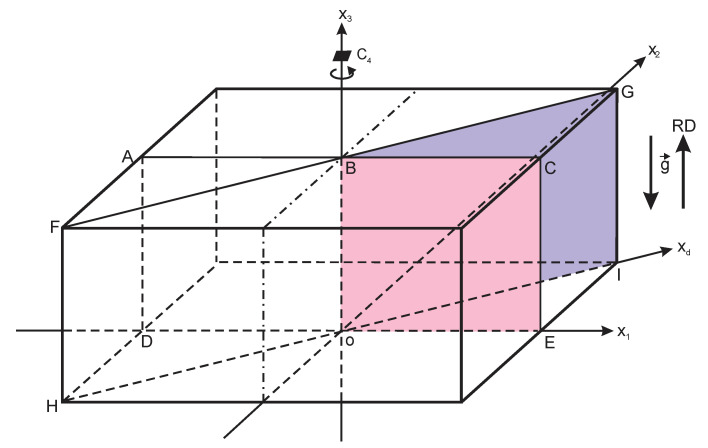
Vertical section planes ACED and FGIH of the PU foam block.

Density distribution in the vertical section plane ACED of the block was considered; Figure 4. The section ACED lies in the x_1_ox_3_ plane and is perpendicular to the sides of the block and the horizontal layers. Due to the reflection symmetry with respect to the ox_3_ axis [31,32], only the first quadrant, BCEo, of section ACED is considered: 0.0 ≤ x_1_ ≤ L_1_/2 mm = 115 mm and 0.0 ≤ x_3_ ≤ L_3_ = 100 mm (further—vertical perpendicular section).

First, density values from the rows x_2_ = 0.0 mm (along the ox_1_ axis) of the six expanded density matrices of the first quadrants of horizontal layers (Tables S12–S17 of the Supplementary Materials) were grouped into a basic density matrix [6 × 21] of the vertical section BCEo (Table S18 of the Supplementary Materials).

Then, number of data points in the basic matrix was increased along ox_3_ axis. The number of partition points was selected such as to ensure similar steps along the ox_1_ and ox_3_ axes: n_3_ = 18 + 1 = 19. The step along ox_3_ axis equals Δx_3_ = L_3_: 18 = 100 mm: 18 = 5.6 mm, Δx_3_ ≈ Δx_1_ = Δx_2_ = 5.8 mm and Δξ_3_ = Δx_3_/L_3_ = 0.056 = 5.6%. Elementary material unit in the x_1_ox_3_ plane is approximately a square. The approximating equations were determined for each column of the basic density matrix as second-degree polynomials

ρ = A_4_(ξ_3_)^2^ + B_4_ξ_3_ + C_4_; (9)

where A_4_, B_4_, and C_4_ are coefficients. Using Equation (9), the expanded density matrix of 21 × 19 = 399 elements was calculated for the vertical perpendicular section (Table S19 of the Supplementary Materials).

#### 2.6.2. Vertical Section in the Plane of Space Diagonals of Block

Density distribution was estimated in the vertical section FGIH, comprising the space diagonals FI and HG of the block (Figure 4). Due to the reflection symmetry with respect to the ox_3_ axis, only one half of the section plane was considered, i.e., the BGIo plane (further—vertical diagonal section). First, density values from the expanded density matrices of the horizontal layers (Tables S12–S17 of the Supplementary Materials) along direction ox_d_ of the face diagonal oI of block were grouped into a basic density matrix [21 × 6] of the vertical section BGIo plane (Table S20 of the Supplementary Materials).

It has to be noticed that coordinate x_1_ of a data point from Table S20 of the Supplementary Materials is a projection of coordinate x_d_ on direction ox_1_:

x_1_ = x_d_ cos45°, then x_d_ = x_1_/cos45° = 0.7071x_1_. (10)

Then, the length of the sides oI and BG of the vertical section BGIo is as follows:

oI = oE/cos∠IoE = oE/0.7071 = 115 mm/0.7071 = 162.6 mm;
(11) BG = oI = L_d_ = 162.6 mm

And the size of the BGIo plane equals 163 mm × 100 mm. Step Δx_d_ along ox_d_ axis, which corresponds to the step Δx_1_ along ox_1_ axis, is calculated as

Δx_d_ = Δx_1_/cos45° = 0.7071Δx_1_ = 0.7071 × 5.75 mm = 8.1 mm. (12)

It can be seen that Δx_d_ > Δx_1_ = Δx_2_ ≈ Δx_3_. To provide an equivalent analysis along all axes, equal steps along the sides of sections have to be ensured horizontally and vertically. Therefore, the basic density matrix has to be recalculated. The density values from Table S20 of the Supplementary Materials were approximated with third-degree polynomials

ρ = A_5_(ξ_d_)^3^ + B_5_(ξ_d_)^2^ + C_5_ξ_d_ + D; (13)

where A_5_, B_5_, C_5,_ and D are coefficients, and ξ_d_ is the relative coordinate along ox_d_. The third-degree polynomials were used as model functions because they ensured the highest value of the coefficient of determination, R^2^. By setting the step Δx_d_ equal to Δx_1_ = Δx_2_ = 5.8 mm, the corresponding number of points was estimated:

Δx_d_ = 5.8 mm; n_d_ = L_d_/Δx_d_ = 28.3 ≈ 28. (14)

Hence, Δξ_d_ = Δx_d_/L_d_ = 5.8 mm/162.6 mm = 3.5%. Using Equation (14), the basic density matrix was recalculated for 28 partition points of the diagonal, L_d_ (Table S21 of the Supplementary Materials).

Then, the number of data points was increased along the ox_3_ axis. The number of points was selected such as to ensure equal steps along the ox_1_ and ox_3_ axes: n_3_ = 18 + 1 = 19. Then, the step along ox_3_ axis equals Δx_3_ = L_3_: 18 = 100 mm: 18 = 5.6 mm, Δx_3_ ≈ Δx_1_ = Δx_2_ = 5.8 mm and Δξ_3_ = Δx_3_/L_3_ = 0.056 = 5.6%, and elementary material unit in the x_1_ox_3_ plane is approximately a square. Approximating equations were determined for each column of Table S21 of the Supplementary Materials as second-degree polynomials:

ρ = A_6_(ξ_3_)^2^ + B_6_ξ_3_ + C_6_; (15)

where A_6_, B_6_, and C_6_—coefficients. Using Equation (15), the expanded density matrix of 21 × 19 = 399 elements was calculated for the vertical diagonal section (Table S22 of the Supplementary Materials).

### 2.7. Density Gradients

Density gradient in the horizontal layers along axis ox_1_ was calculated from the expanded density matrices in Tables S12–S17 of Supplementary Materials as

(16) $$\mathrm{grad}\rho (\xi_{1})=\frac{\Delta \rho (\xi_{1})}{\Delta \xi_{1}},\;\mathrm{where}\;\xi_{1}=0.058.$$

due to rotational symmetry gradρ(ξ_2_) = gradρ(ξ_1_). Gradients in the vertical perpendicular section along ox_1_ and ox_3_ were calculated from the expanded density matrix in Table S19 of the Supplementary Materials as

(17) $$\mathrm{grad}\rho (\xi_{1})\;\approx \;\frac{\Delta \rho (\xi_{1})}{\Delta \xi_{1}}\mathrm{and}\;\mathrm{grad}\rho (\xi_{3})\;\approx \;\frac{\Delta \rho (\xi_{3})}{\Delta \xi_{3}},$$

where Δξ_1_ = 0.058 and Δξ_3_ = 0.056. Gradients in the vertical diagonal section along ox_d_ and ox_3_ were calculated from the expanded density matrix in Table S22 of the Supplementary Materials as

(18) $$\mathrm{grad}\rho (\xi_{d})\;\approx \;\frac{\Delta \rho (\xi_{d})}{\Delta \xi_{d}}\mathrm{and}\;\mathrm{grad}\rho (\xi_{3})\;\approx \;\frac{\Delta \rho (\xi_{3})}{\Delta \xi_{3}},$$

where Δξ_3_ = 0.056 and Δξ_d_ = 0.035. The gradients were calculated in kg/m^3^ per 1 cm.

### 2.8. Density Charts and Graphs

Density distribution charts were constructed for the first quadrant of six horizontal layers and two vertical sections. The range of calculated densities, 86–116 kg/m^3^, was divided into 15 zones of width Δρ = 2 kg/m^3^. Zones were numbered from center to periphery of a section, and each was assigned its own color (Table 6). Dividing into narrower zones would yield unreliable results, since width Δρ < 2 kg/m^3^ is comparable to the expanded uncertainty, 1.2 kg/m^3^ ≤ U_95%_ ≤ 1.5 kg/m^3^, of the density measurements.

Elementary material units Δx_1_ × Δx_2_, Δx_1_ × Δx_3_, and Δx_d_ × Δx_3_ of the expanded density matrices (Tables S12–S17, S19, and S21 of the Supplementary Materials) were each assigned a color corresponding to the density value. Equidensity lines (by analogy with the equipotential lines of electric field), connecting units of equal density, were marked on the charts. In 3D space, equidensity lines form equidensity surfaces. The density charts were provided with three columns on the left and three rows at the bottom, containing ordinal numbers, as well as absolute and relative coordinates of the material units. This allows the dimensions of the density zones to be determined. Characteristics of the density zones were determined and analyzed.

For better visualization, graphs of density and its gradients in horizontal layers and both vertical sections were drawn, depending on one coordinate, at several constant values of the other, e.g., the dependence of gradient in the vertical diagonal section on ξ_d_ at ξ_3_ = 0.0, 0.22, 0.44, 0.67, 0.89, and 1.00.

## 3. Results and Discussion

### 3.1. Microscopy Data 

The average anisotropy degree for the foams in the plane of a slice, which is parallel to (1) x_1_ox_2_, (2) x_1_ox_3_, or (3) x_2_ox_3_ planes, is given in Table 7. In all planes of the samples, AD_av_ ≈ 1.0, indicating a local uniform spatial distribution of the polymeric struts. However, the distribution of density is inhomogeneous, as demonstrated by the density charts below. As the foams’ density increases, the length of the struts decreases, the cross-section increases, and the size of the polymeric nodes increases [6,7,8]. For rigid PU foams manufactured in a sealed mold, at overpressure, uniform local spatial distribution of the polymeric struts exists alongside with graded density [33,34].

### 3.2. Distribution of Density

Density distribution charts of the first quadrants of the horizontal layers are shown in Figures S1–S3 of the Supplementary Materials. It is concluded from the density charts that the central density zone of all layers, Nos. 1–6, is a circle with a center at the origin, “o”, of coordinate system x_1_ox_2_; adjacent zones are circular rings with the same center. For the circular zones, the dimensions along axes ox_1_ and ox_2_ are equal: x_1_ = x_2_. In direction to the sides of the block, the outer shape of the zones tends towards a square with rounded corners due to influence of the rectangular mold. As the foaming liquid mixture expands, it fills the working volume of the mold and mirrors the square shape of its cross-section.

Density is the lowest in the central circle and highest in the corners of layers. The lowest calculated density was identified in layer No. 4 (86 kg/m^3^ in the center of central zone), and the highest in layer No. 1 (109 kg/m^3^ in the corners). The experimental value of the lowest density equals 86.9 kg/m^3^ (sample No. 4 in characteristic column No. 6; Table 5) and of the highest—107.3 kg/m^3^ (sample No. 1 in column No. 1; Table 5). The corresponding relative differences between the calculated and experimental values are 1.0% and 1.6%. In layers No. 4 and No. 5, the density distribution is similar; however, the central circular zone of layer No. 4 has a bigger radius than that of layer No. 5.

**Figure 5:**
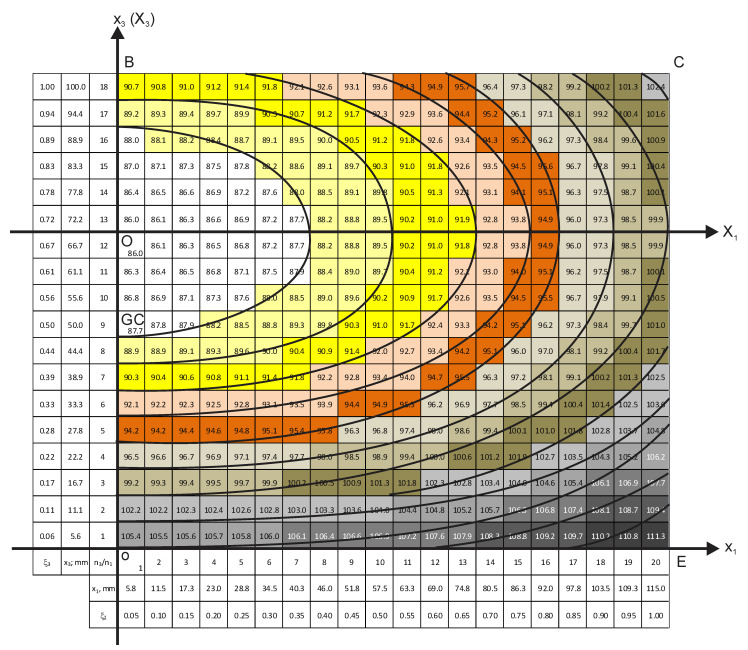
The chart of density distribution in the vertical perpendicular section, x_1_ox_3_; X_1_OX_3_—the local coordinate system.

**Figure 6:**
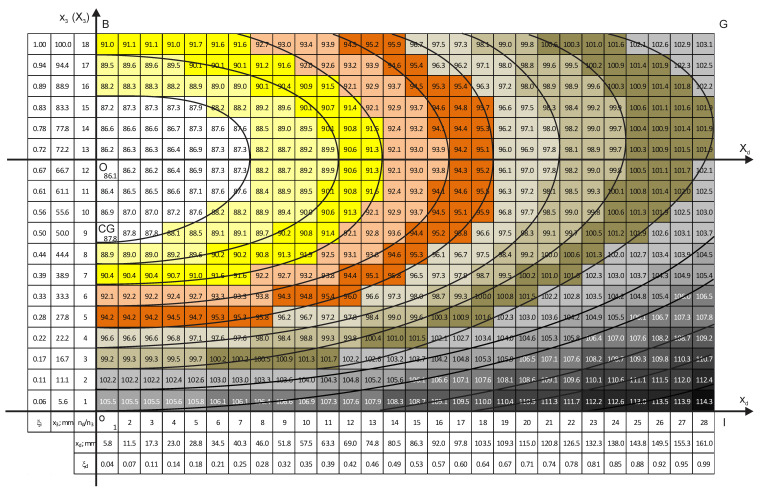
The chart of density distribution in the vertical diagonal section, x_d_ox_3_; X_d_OX_3_—the local coordinate system.

Density distribution charts of the two vertical sections are given in Figure 5 and Figure 6.

The density charts show that all equidensity lines have a common center, which is further denoted as the block’s foaming center. The foaming center corresponds to the lowest calculated density, 86.0 kg/m^3^. In order to operate with the concentric equidensity lines, two local coordinate systems are introduced: (1) the X_1_OX_3_ system, which lies in the plane of the vertical perpendicular section x_1_ox_3_ (Figure 5), and (2) the X_d_OX_3_ system in the plane of the vertical diagonal section x_d_ox_3_ (Figure 6). Both systems are positioned with their origin, “O”, in the foaming center.

It is of theoretical and practical interest to determine location of the foaming center along the height of an untrimmed block. In the trimmed block, point “O” is located between elementary material volumes with coordinates x_3_ = 66.7 mm and x_3_ = 72.2 mm (ξ_3_ ≈ 0.67 and ξ_3_ ≈ 0.72). The coordinate ξ_3_ ≈ 0.67 corresponds to the surface between layers No. 4 and No. 5 (x_3_ = 16.7 mm × 4 layers ≈ 66.8 mm; Figure 5 and Figure 6). Since ≈15 mm was removed from the top and bottom of the block during trimming, the distance from “O” to the bottom of the untrimmed block is x_3b_ = 67 mm + 15 mm = 82 mm, and from “O” to the top of an untrimmed block, it is x_3t_ = 33 mm + 15 mm = 48 mm. That yields ratios

η_t_ = x_3t_/L_30_ ≈ 0.37 and η_b_ = x_3t_/L_30_ ≈ 0.63, (19)

where L_30_ = 130 mm is the height of an untrimmed block. The point “O” lies Δ = 82 mm − 65 mm = 17 mm above the geometric center “GC” (L_30_/2 = 65 mm) of an untrimmed block (Figure 5 and Figure 6). The ratios η_t_ and η_b_ should be determined at other technological parameters of manufacturing of the block as well.

It has to be proved that the equidensity lines No. 1–7 (density < 100 kg/m^3^) in the vertical perpendicular section are ellipses. An equidensity line intersects the axis OX_1_ and OX_3_ at certain points, “a” and “b” (Figure 5). If the line is an ellipse, its points (X_1_, X_3_) have to satisfy the canonical equation of an ellipse:

(20) $$\frac{X_{1}^{2}}{{\left(a_{i}\right)}^{2}}+\frac{X_{3}^{2}}{{\left(b_{i}\right)}^{2}}=1.0;\;1,\;2,\;\ldots \;,\;7.$$

Arbitrary points of the equidensity line were selected, and their coordinates, X_1_ and X_3_, were determined from the density charts with an uncertainty, proportional to the dimensions of the material units Δx_3_ ≈ Δx_1_ = 5.8 mm. Then, X_1_ and X_3_ were substituted into Equation (20). It was identified that within the mentioned uncertainties, the points of equidensity lines No. 1–7 fulfil Equation (20), which proves that the lines are ellipses.

Semi-axes a and b of the elliptical density zones No. 1–7 were determined from the density chart of the vertical perpendicular section (Figure 5) as the coordinates of intersection of the outer equidensity line of a density zone with the OX_1_ and OX_3_ axes [35]. Then, the linear eccentricity (c) and eccentricity (e) were calculated: $c=\sqrt{a^{2}-b^{2}}$ and e = $\sqrt{1-\;\frac{b^{2}}{a^{2}}}$ (Table 8). The ratio of average semi-axes and average eccentricity were calculated as

(21) $$\frac{\bar{b}}{\bar{a}}=0.48\;\pm \;0.035\;(7\%)\;\mathrm{and}\;\bar{e}\;=0.87\;\pm \;0.02\;(2\%).$$

for the zones No. 1–7, which identifies similar characteristics of the equidensity lines and density zones.

For equidensity lines No. 8–12 (ρ ≥ 100 kg/m^3^), the points “a” and “b” cannot be determined from the density chart in Figure 5, because intersections of the lines with the OX_1_ and OX_3_ axes lie outside the chart. The available segments of the equidensity lines No. 8–12 are insufficient to assess whether the lines are ellipses.

In the vertical diagonal section (Figure 6), the equidensity lines are similar (elliptical) to those in the vertical perpendicular section in the center of the mold, up to densities ≈91 kg/m^3^ (equidensity lines No. 1–3). At higher densities, the equidensity lines are distorted; however, they still have a common center. The distortion may be caused by the fact that the diagonal FG of the mold’s cross-section is longer than the perpendicular AC to the wall of the mold (Figure 4). The foaming mixture has more space to expand in the diagonal direction than in the perpendicular one, which distorts the elliptical shape. In the central part of the mold, the influence of the mold’s cross-section is the smallest.

Influence of the mold’s corners is evident as well: in the lower right corner of the chart (Figure 6), the density is highest of all: ≈114 kg/m^3^. The corners of the rectangular parallelepiped block have three contact surfaces with the relatively cooler mold. Compared to the other points in the block, heat exchange at the corners is the highest, which leads to slower blowing (gas generation), premature curing, and higher foam densities [5,6,7,8]. The density distribution is influenced by the Earth’s gravity as well, when the less dense material units rise in the effective medium of denser units under action of buoyancy. It can be expected that under microgravity conditions, without acceleration, the foaming center will coincide with the geometric center of the block.

Graphs of density and its gradients in the six horizontal and two vertical sections are given in Figures S4–S13 of Supplementary Materials in dependence of one coordinate of the section plane at several fixed values of the other. It can be seen that density changes nonlinearly in the horizontal, as well as vertical sections of the block. The most complex is variation in density along diagonal axis ox_d_, which is approximated by a third-degree polynomial. All gradients except the one along diagonal axis ox_d_ vary according to linear rules. Gradient of density in the vertical diagonal section depending on coordinate ξ_d_ at fixed values of coordinate ξ_3_ is nonlinear. Gradients depending on coordinate ξ_3_ change sign from “−” to “+”.

## 4. Conclusions

(1) Approximating the experimental data with second- and third-degree polynomials, a mathematical model was elaborated for distribution of density and its gradients in a rigid block of polyurethane foams, manufactured in a sealed rectangular mold.
(2) The results of numerical calculations—the density charts—showed that in the horizontal layers, the density zones and equidensity lines are circular and square with rounded corners. In the vertical perpendicular section, the zones are circular and elliptical, while in the vertical diagonal section, there are circular, elliptical, and distorted zones as well. The rectangular, parallelepiped shape of the mold influences the shape of density zones.
(3) It was determined that the foaming center is located ≈ 17 mm above the geometric center of untrimmed block. The asymmetry may have occurred at the stage when the chemical composition of PU foams was still liquid and central, less dense units rose in the efficient media of denser units under the force of buoyancy (like bubbles rising in a liquid). Further studies should deal with determining the of location of the foaming center at other values of the parameters of PU foams’ manufacturing technology (the size and proportions of the sealed mold, the chemical formulation, overpressure, etc.).

## Figures and Tables

**Table 1:** Composition for production of rigid PU foams.

Number	Component	Trade Name	Producer	pbw
1	Polyether polyol	Lupranol 3000	BASF SE (Ludwigshafen, Germany)	45
2	Polyester polyol	Lupraphen 1901/1		30
3	Chain extender	Diethylene glycol	Sigma-Aldrich Co. LLC (St. Louis, MO, USA)	25
4	Flame retardant	Tris(1-chloro-2-propyl) phosphate	Albemarle Corp. (Charlotte, NC, USA)	15
5	Surfactant	Silicone L6915LV	Momentive Performance Materials (Niskayuna, NY, USA)	1.5
6	Catalyst package	PC CAT NP10	Air Products and Chemicals Inc. (Allentown, PA, USA)	0.3
7	Catalyst package	Kosmos 19	Evonik Industries AG (Essen, Germany)	0.3
8	Physical blowing agent	Pentane	Sigma-Aldrich Co. LLC (St. Louis, MO, USA)	25
9	Chemical blowing agent	Distilled water		0.1
10	Polyisocyanate	Desmodur 44V20L	Covestro AG (Leverkusen, Germany)	168

**Table 2:** The coordinates of the geometric centers of microscopy samples.

Number of Layer	Number of Sample	Coordinates of Geometric Centers; mm		
		x_1_	x_2_	x_3_
2	1	0.0	0.0	25.5
	2	57.5	0.0	25.5
5	1	0.0	0.0	76.5
	2	57.5	0.0	76.5

**Table 3:** Features of characteristic locations of columns in the block.

Kind (j) of Location	Description of Location	Number of Columns	Ordinal Numbers of Columns	Number of Contact Surfaces with the Mold
1	Corner	4	1, 5, 21, and 25	4
2	Next to the corner	8	2, 4, 6, 10, 16, 20, 22, and 24	3
3	Center of side of the block	4	3, 11, 15, and 23	3
4	On diagonal of cross-section of the block	4	7, 9, 17, and 19	2
5	Between diagonal locations	4	8, 12, 14, and 18	2
6	Center of the block	1	13	2

**Table 4:** Average density of samples in characteristic columns (±σ is standard deviation, and v is coefficient of variation, in %).

Number (i) of a Sample	Average Density, ρ_av_; kg/m^3^					
	Kind j of Location of a Characteristic Column					
	1	2	3	4	5	6
6	100.6 ±1.1 (1.1%)	98.1 ±0.7 (0.7%)	97.9 ±0.2 (0.2%)	93.4 ±0.6 (0.6%)	92.3 ±0.8 (0.8%)	89.2 -
5	100.0 ±0.8 (0.8%)	97.1 ±1.1 (1.1%)	96.7 ±0.3 (0.3%)	92.2 ±0.5 (0.5%)	89.2 ±0.3 (0.3%)	88.8 -
4	99.7 ±0.6 (0.6%)	96.2 ±0.9 (0.9%)	95.6 ±0.8 (0.8%)	90.6 ±0.3 (0.3%)	88.4 ±0.5 (0.6%)	87.5 -
3	100.5 ±0.4 (0.4%)	96.3 ±1.1 (1.1%)	97.0 ±0.3 (0.3%)	91.4 ±0.7 (0.8%)	89.9 ±0.6 (0.6%)	88.3 -
2	101.5 ±1.0 (1.0%)	98.9 ±0.7 (0.7%)	98.8 0.3 (0.3%)	93.9 ±0.1 (0.1%)	92.3 ±0.6 (0.6%)	89.9 -
1	108.1 ±0.8 (0.7%)	105.7 ±0.9 (0.9%)	105.7 ±1.0 (0.9%)	102.3 ±0.9 (0.9%)	102.3 ±1.3 (1.2%)	98.6 -

**Table 5:** The fitted average density of samples in characteristic columns.

Number (i) of a Sample	Average Density, ρ_av_; kg/m^3^					
	The Kind (j) of a Characteristic Column					
	1	2	3	4	5	6
6	101.1	98.8	98.4	94.2	92.7	90.2
5	99.4	96.2	96.0	91.0	88.9	87.5
4	99.1	95.6	95.5	90.2	87.8	86.9
3	100.4	96.8	96.8	91.6	89.6	88.4
2	103.1	100.0	99.9	95.3	94.1	91.9
1	107.3	105.0	104.9	101.4	101.4	97.5

**Table 6:** The colour legend of density zones.

Number of a Zone	1	2	3	4	5	6	7	8
Density range; kg/m^3^	86–88	88–90	90–92	92–94	94–96	96–98	98–100	100–102
Color								
Number of a zone	9	10	11	12	13	14	15	
Density range; kg/m^3^	102–104	104–106	106–108	108–110	110–112	112–114	114–116	
Color								

**Table 7:** The density and average anisotropy degree of the PU foams (±standard uncertainty).

Number of Layer	Number of Sample	Density; kg/m^3^	AD_12av_	AD_13av_	AD_23av_
			(x_1_ox_2_ Plane)	(x_1_ox_3_ Plane)	(x_2_ox_3_ Plane)
2	1	93.5	1.00 ± 0.02	0.99 ± 0.02	0.99 ± 0.02
	2	95.2	1.01 ± 0.01	1.00 ± 0.02	1.01 ± 0.02
5	1	86.0	1.00 ± 0.02	0.95 ± 0.03	0.96 ± 0.03
	2	90.5	1.01 ± 0.01	0.99 ± 0.02	0.98 ± 0.02

**Table 8:** Parameters of elliptical density zones No. 1–7 in the vertical perpendicular section.

N_dz_	Range of Density; kg/m^3^	Average Density ρ_av_; kg/m^3^	Semi-Axis a; mm	Semi-Axis b; mm	Ratio b/a	Linear Eccentricity c; mm	Eccentricity e = c/a
1	86–88	87	40.3	22.2	0.55	33.6	0.83
2	88–90	89	57.5	27.8	0.48	50.3	0.88
3	90–92	91	74.8	33.3	0.45	66.9	0.90
4	92–94	93	86.3	38.9	0.45	77.0	0.89
5	94–96	95	92.0	44.4	0.48	80.6	0.88
6	96–98	97	103.5	50.0	0.48	90.6	0.88
7	98–100	99	115.0	55.6	0.48	100.7	0.88

## Data Availability

The raw data supporting the conclusions of this article will be made available by the authors upon request.

## Supplementary Materials

The following supporting information can be downloaded at https://www.mdpi.com/article/10.3390/polym18060733/s1, Table S1. Density of samples, Coordinates of the geometric centers of the microscopy samples, Table S2. Basic density matrix of the block, Table S3. Absolute coordinates x_1_ and x_2_ of the centres of the 25 uncut samples of a horizontal layer (In millimetres), Table S4. Relative coordinates ξ_1_ and ξ_2_ of the centres of the 25 uncut samples of a horizontal layer, Table S5. Absolute and relative coordinates x_3_ and ξ_3_ of the centres of samples in the horizontal layers № 1–6, Table S6. The basic density matrix of the horizontal layer № 1, Table S7. The averaged basic density matrix of the horizontal layer № 2, Table S8. The averaged basic density matrix of the horizontal layer № 3, Table S9. The averaged basic density matrix of the horizontal layer № 4, Table S10. The averaged basic density matrix of the horizontal layer № 5, Table S11. The averaged basic density matrix of the horizontal layer № 6, Table S12. The expanded density matrix of horizontal layer № 1 (x_1_ and x_2_ – coordinates, in millimetres; ξ_1_ and ξ_2_ – relative coordinates, and k_1_ and k_2_ – numbers of columns and rows), Table S13. The expanded density matrix of horizontal layer № 2 (x_1_ and x_2_ – coordinates, in millimetres; ξ_1_ and ξ_2_ – relative coordinates, and k_1_ and k_2_ – numbers of columns and rows), Table S14. The expanded density matrix of horizontal layer № 3 (x_1_ and x_2_ – coordinates, in millimetres; ξ_1_ and ξ_2_ – relative coordinates, and k_1_ and k_2_ – numbers of columns and rows), Table S15. The expanded density matrix of horizontal layer № 4 (x_1_ and x_2_ – coordinates, in millimetres; ξ_1_ and ξ_2_ – relative coordinates, and k_1_ and k_2_ – numbers of columns and rows), Table S16. The expanded density matrix of horizontal layer № 5 (x_1_ and x_2_ – coordinates, in millimetres; ξ_1_ and ξ_2_ – relative coordinates, and k_1_ and k_2_ – numbers of columns and rows), Table S17. The expanded density matrix of horizontal layer № 6 (x_1_ and x_2_ – coordinates, in millimetres; ξ_1_ and ξ_2_ – relative coordinates, and k_1_ and k_2_ – numbers of columns and rows), Table S18. The basic density matrix in vertical perpendicular section (x_1_ and x_3_ – coordinates, in millimetres; ξ_1_ and ξ_3_ – relative coordinates, and n_1_ and n_3_ – numbers of columns and rows), Table S19. The expanded density matrix in vertical perpendicular section (x_1_ and x_3_ – coordinates, in millimetres; ξ_1_ and ξ_3_ – relative coordinates, and n_1_ and n_3_ – numbers of columns and rows), Table S20. The basic density matrix in vertical diagonal section (x_1,_ x_3_, and x_d_ – coordinates, in millimetres; ξ_1_, ξ_3_, and ξ_d_ – relative coordinates, and n_d_ and n_3_ – numbers of columns and rows), Table S21. The basic density matrix for vertical diagonal section, columns № 0 – 14, at Δx_d_ = Δx_1_ = Δx_2_ = 5.8 mm (x_3_ and x_d_ – coordinates, in millimetres; ξ_3_ and ξ_d_ – relative coordinates, and n_d_ and n_3_ – numbers of columns and rows), Table S21 (continuation). The basic density matrix for vertical diagonal section, columns № 15 – 28, at Δx_d_ = Δx_1_ = Δx_2_ = 5.8 mm (x_3_ and x_d_ – coordinates, in millimetres; ξ_3_ and ξ_d_ – relative coordinates, and n_d_ and n_3_ – numbers of columns and rows), Table S22. The expanded density matrix in vertical diagonal section, columns № 0 - 14 (x_3_ and x_d_ – coordinates, in millimetres; ξ_3_ and ξ_d_ – relative coordinates, and n_d_ and n_3_ – numbers of columns and rows), Table S22 (continuation). The expanded density matrix in vertical diagonal section, columns № 15 - 28 (x_3_, and x_d_ – coordinates, in millimetres; ξ_3_ and ξ_d_ – relative coordinates, and n_d_ and n_3_ – numbers of columns and rows), Figure S1. Charts of density distribution in the horizontal layers a) № 1 and b) № 2; parallel to the plane x_1_ox_2_, Figure S2. Charts of density distribution in the horizontal layers a) № 3 and b) № 4; parallel to the plane x_1_ox_2_, Figure S3. Charts of density distribution in the horizontal layers a) № 5 and b) № 6; parallel to the plane x_1_ox_2_, Figure S4. Density depending on coordinate ξ_1_ at fixed values of coordinate ξ_2_ 1) ξ_2_ = 0.0 (Black), 2) ξ_2_ = 0.2 (Grey), 3) ξ_2_ = 0.4 (Violet), 4) ξ_2_ = 0.6 (Blue), 5) ξ_2_ = 0.8 (Red), and 6) ξ_2_ = 1.0 (Brown); a) – f) horizontal layers № 1 – 6, Figure S5. Density gradient depending on coordinate ξ_1_ at fixed values of coordinate ξ_2_ 1) ξ_2_ = 0.0 (Black), 2) ξ_2_ = 0.2 (Grey), 3) ξ_2_ = 0.4 (Violet), 4) ξ_2_ = 0.6 (Blue), 5) ξ_2_ = 0.8 (Red), and 6) ξ_2_ = 0.95 (Brown); a) – f) horizontal layers № 1 – 6, Figure S6. Density in the vertical perpendicular section depending on coordinate ξ_1_ at fixed values of coordinate ξ_3_ 1) ξ_3_ = 0.00 (Black), 2) ξ_3_ = 0.22 (Grey), 3) ξ_3_ = 0.44 (Violet), 4) ξ_3_ = 0.67 (Blue), 5) ξ_3_ = 0.89 (Red), and 6) ξ_3_ = 1.00 (Brown), Figure S7. Density in the vertical perpendicular section depending on coordinate ξ_3_ at fixed values of coordinate ξ_1_ 1) ξ_1_ = 0.0 (Black), 2) ξ_1_ = 0.2 (Grey), 3) ξ_1_ = 0.4 (Violet), 4) ξ_1_ = 0.6 (Blue), 5) ξ_1_ = 0.8 (Red), and 6) ξ_1_ = 1.00 (Brown), Figure S8. Density gradient in the vertical perpendicular section depending on coordinate ξ_1_ at fixed values of coordinate ξ_3_ 1) ξ_3_ = 0.0 (Black), 2) ξ_3_ = 0.22 (Grey), 3) ξ_3_ = 0.44 (Violet), 4) ξ_3_ = 0.67 (Blue), 5) ξ_3_ = 0.89 (Red), and 6) ξ_3_ = 1.00 (Brown), Figure S9. Density gradient in the vertical perpendicular section depending on coordinate ξ_3_ at fixed values of coordinate ξ_1_ 1) ξ_1_ = 0.0 (Black), 2) ξ_1_ = 0.2 (Grey), 3) ξ_1_ = 0.4 (Violet), 4) ξ_1_ = 0.6 (Blue), 5) ξ_1_ = 0.8 (Red), and 6) ξ_1_ = 1.0 (Brown), Figure S10. Density in the vertical diagonal section in dependence of coordinate ξ_d_ at fixed values of coordinate ξ_3_ 1) ξ_3_ = 0.0 (Black), 2) ξ_3_ = 0.22 (Grey), 3) ξ_3_ = 0.44 (Violet), 4) ξ_3_ = 0.67 (Blue), 5) ξ_3_ = 0.89 (Red), and 6) ξ_3_ = 1.00 (Brown), Figure S11. Density in the vertical diagonal section in dependence of coordinate ξ_3_ at fixed values of coordinate ξ_d_ 1) ξ_d_ = 0.0 (Black), 2) ξ_d_ = 0.21 (Grey), 3) ξ_d_ = 0.42 (Violet), 4) ξ_d_ = 0.64 (Blue), 5) ξ_d_ = 0.85 (Red), and 6) ξ_d_ = 1.00 (Brown), Figure S12. Density gradient in the vertical diagonal section depending on coordinate ξ_d_ at fixed values of coordinate ξ_3_ 1) ξ_3_ = 0.0 (Black), 2) ξ_3_ = 0.22 (Grey), 3) ξ_3_ = 0.44 (Violet), 4) ξ_3_ = 0.67 (Blue), 5) ξ_3_ = 0.89 (Red), and 6) ξ_3_ = 1.00 (Brown), Figure S13. Density gradient in the vertical diagonal section depending on coordinate ξ_3_ at fixed values of coordinate ξ_d_ 1) ξ_d_ = 0.0 (Black), 2) ξ_d_ = 0.2 (Grey), 3) ξ_d_ = 0.4 (Violet), 4) ξ_d_ = 0.6 (Blue), 5) ξ_d_ = 0.8 (Red), and 6) ξ_d_ = 1.00 (Brown).
