# Supplementary material for: Estimation of Density Distribution in a Rigid PU Foam Block Manufactured in a Sealed Mold
Source: Polymers (Basel). 2026 Mar 17;18(6):733. doi: 10.3390/polym18060733 (PMC13030234; doi:10.3390/polym18060733)
Supplement: Supplementary file 1 [file polymers-18-00733-s001.zip › polymers-4160529-supplementary.pdf]

Supplementary Materials to the paper

Estimation of Density Distribution in a Rigid PU Foam Block Manufactured in a Sealed Mold

Ilze Beverte, Ugis Cabulis and Jānis Andersons

S1. Density of samples

Table S1. Density of samples.

Number of a sample	Density of samples ρ_n; kg/m³				
	Ordinal number of a column				
	1	2	3	4	5
6	101.6	98.8	97.7	96.8	101.1
5	99.5	97.8	96.6	97.7	99.4
4	100.3	97.3	96.7	96.3	99.8
3	101.0	96.4	97.2	96.7	100.0
2	100.8	99.5	99.0	98.1	100.9
1	108.5	107.4	106.9	105.0	108.8
	6	7	8	9	10
6	98.9	94.0	91.6	93.7	97.8
5	98.7	92.1	89.3	91.6	95.9
4	96.5	90.4	87.9	90.5	95.0
3	98.1	90.8	89.4	90.9	94.3
2	100.0	93.8	93.1	94.0	98.6
1	105.9	102.5	100.6	101.0	105.9
	11	12	13	14	15
6	97.8	93.4	89.2	92.0	98.1
5	97.1	88.8	88.8	89.4	96.5
4	94.8	89.1	87.5	88.3	95.4
3	96.5	89.6	88.3	90.7	97.2
2	98.4	92.2	89.9	91.7	99.0
1	104.6	102.6	98.6	103.6	105.5
	16	17	18	19	20
6	97.7	92.6	92.3	93.4	98.0
5	97.0	92.8	89.2	92.2	97.4
4	95.6	91.0	88.4	90.6	97.3
3	96.5	92.4	89.9	91.4	96.6
2	98.5	93.9	92.3	93.9	99.3
1	104.7	103.3	102.3	102.3	104.5
	21	22	23	24	25
6	99.0	98.7	97.9	98.1	100.6

https://doi.org/10.3390/polym18060733

5	101.1	95.2	96.7	97.1	100.0
4	98.9	95.1	95.6	96.2	99.7
3	100.4	95.7	97.0	96.3	100.5
2	102.9	98.2	98.8	98.9	101.5
1	107.0	106.3	105.7	105.7	108.1

Coordinates of the geometric centers of the microscopy samples

1) The geometric center of a rectangular parallelepiped is defined as the intersection point of its four spatial diagonals.
2) The four microscopy samples are cut as rectangular parallelepipeds with their geometric centers located in the x_1ox_3 plane, therefore the corresponding coordinates equal $x_2 = 0.0$ mm.
3) The geometric centers of samples № 1 from layers № 2 and № 5 are located on the ox_3 axis, therefore, the corresponding coordinates are $x_1 = 0.0$ mm.
4) The geometric centers of samples № 2 from layers № 2 and № 5 are located on the ox_1 axis, midway between the origin "o" of the $x_1ox_2x_3$ coordinate system and the side of the block, therefore corresponding coordinate is $x_1 = 57.5$ mm.
5) The coordinate x_3 of the geometric centers of samples is calculated by adding a half the height of the sample to the height of a) layer № 1 for the samples from layer № 2 and b) layers № 1 – № 4 for the samples from layer № 5, hence the corresponding values $x_3 = 25.2$ mm and $x_3 = 76.5$ mm.

Table S2. Basic density matrix of the block.

Number of a sample	Density of samples ρ_n; kg/m^3				
	Ordinal number of a column				
	1	2	3	4	5
6	101.1	98.8	98.4	98.8	101.1
5	99.4	96.2	96.0	96.2	99.4
4	99.1	95.6	95.5	95.6	99.1
3	100.4	96.8	96.8	96.8	100.4
2	103.1	100.0	99.9	100.0	103.1
1	107.3	105.0	104.9	105.0	107.3
	6	7	8	9	10
6	98.8	94.2	92.7	94.2	98.8
5	96.2	91.0	88.9	91.0	96.2
4	95.6	90.2	87.8	90.2	95.6
3	96.8	91.6	89.6	91.6	96.8
2	99.9	95.3	94.1	95.3	99.9
	11	12	13	14	15
1	104.9	101.4	101.4	101.4	104.9
6	98.4	92.7	90.2	92.7	98.4

5	96.0	88.9	87.5	88.9	96.0
4	95.5	87.8	86.9	87.8	95.5
3	96.8	89.6	88.4	89.6	96.8
2	100.0	94.1	91.9	94.1	100.0
1	105.0	101.4	97.5	101.4	105.0
	16	17	18	19	20
6	98.8	94.2	92.7	94.2	98.8
5	96.2	91.0	88.9	91.0	96.2
4	95.6	90.2	87.8	90.2	95.6
3	96.8	91.6	89.6	91.6	96.8
2	99.9	95.3	94.1	95.3	99.9
1	104.9	101.4	101.4	101.4	104.9
	21	22	23	24	25
6	101.1	98.8	98.4	98.8	101.1
5	99.4	96.2	96.0	96.2	99.4
4	99.1	95.6	95.5	95.6	99.1
3	100.4	96.8	96.8	96.8	100.4
2	103.1	100.0	99.9	100.0	103.1
1	107.3	105.0	104.9	105.0	107.3

S2. Absolute and relative coordinates

Table S3. Absolute coordinates x_1 and x_2 of the centres of the 25 uncut samples of a horizontal layer (In millimetres).

Number of a row	Number of a column				
	1	2	3	4	5
1	(– 92; 92)	(– 46, 92)	(0; 92)	(46; 92)	(92; 92)
2	(– 92; 46)	(– 46; 46)	(0; 46)	(46; 46)	(92; 46)
3	(– 92; 0)	(– 46; 0)	(0; 0)	(46; 0)	(92; 0)
4	(– 92; - 46)	(– 46; -46)	(0; – 46)	(46; – 46)	(92; – 46)
5	(– 92; - 92)	(– 46; -92)	(0; – 92)	(46; – 92)	(92; – 92)

Table S4. Relative coordinates ξ_1 and ξ_2 of the centres of the 25 uncut samples of a horizontal layer.

Number of a row	Number of a column				
	1	2	3	4	5
1	(– 0.8; 0.8)	(– 0.4; 0.8)	(0.0; 0.8)	(0.4; 0.8)	(0.8; 0.8)
2	(– 0.8; 0.4)	(– 0.4; 0.4)	(0.0; 0.4)	(0.4; 0.4)	(0.8; 0.4)
3	(– 0.8; 0.0)	(– 0.4; 0.0)	(0.0; 0.0)	(0.4; 0.0)	(0.8; 0.0)
4	(– 0.4; – 0.4)	(– 0.4; – 0.4)	(0.0; – 0.4)	(0.4; – 0.4)	(0.8; – 0.4)
5	(– 0.8; – 0.8)	(– 0.4; – 0.8)	(0.0; – 0.8)	(0.4; – 0.8)	(0.8; – 0.8)

https://doi.org/10.3390/polym18060733

Table S5. Absolute and relative coordinates x_3 and ξ_3 of the centres of samples in the horizontal layers № 1 - 6.

Number of a layer l	Absolute coordinate x_3; mm	Relative coordinate ξ_3
6	91.9	0.92
5	75.2	0.75
4	58.5	0.58
3	41.8	0.42
2	25.1	0.25
1	8.4	0.08

S3.The basic density matrices of the horizontal layers

Table S6. The **basic** density matrix of the horizontal layer № 1.

Number of a row in a layer	Relative co-ordinate ξ_2	Average density ρ; kg/m³				
		Number of the column in a layer m				
		1	2	3	4	5
		Relative coordinate ξ_1				
		– 0.8	– 0.4	0.0	0.4	0.8
1	0.8	107.3	105.0	104.9	105.0	107.3
2	0.4	105.0	101.4	101.4	101.4	105.0
3	0.0	104.9	101.3	97.5	101.3	104.9
4	– 0.4	105.0	101.4	101.4	101.4	105.0
5	– 0.8	107.3	105.0	104.9	105.0	107.3

Table S7. The averaged **basic** density matrix of the horizontal layer № 2.

Number of a row in a layer	Relative coordinate ξ_2	Average density ρ; kg/m³				
		Number of the column in a layer m				
		1	2	3	4	5
		Relative coordinate ξ_1				
		– 0.8	– 0.4	0.0	0.4	0.8
1	0.8	103.1	100.0	99.9	100.0	103.1
2	0.4	100.0	95.3	94.1	95.3	100.0
3	0.0	99.9	94.1	91.9	94.1	99.9
4	– 0.4	100.0	95.3	94.1	95.3	100.0
5	– 0.8	103.1	100.0	99.9	100.0	103.1

https://doi.org/10.3390/polym18060733

Table S8. The averaged **basic** density matrix of the horizontal layer № 3.

Number of a row in a layer	Relative coordinate ξ_2	Average density ρ; kg/m³				
		Number of the column in a layer m				
		1	2	3	4	5
		Relative coordinate ξ_1				
		– 0.8	– 0.4	0.0	0.4	0.8
1	0.8	100.4	96.8	96.8	96.8	100.4
2	0.4	96.8	91.6	89.6	91.6	96.8
3	0.0	96.8	89.6	88.4	89.6	96.8
4	– 0.4	96.8	91.6	89.6	91.6	96.8
5	– 0.8	100.4	96.8	96.8	96.8	100.4

Table S9. The averaged **basic** density matrix of the horizontal layer № 4.

Number of a row in a layer	Relative coordinate ξ_2	Average density ρ; kg/m³				
		Number of the column in a layer m				
		1	2	3	4	5
		Relative coordinate ξ_1				
		– 0.8	– 0.4	0.0	0.4	0.8
1	0.8	99.1	95.6	95.5	95.6	99.1
2	0.4	95.6	90.2	87.8	90.2	95.6
3	0.0	95.5	87.8	86.9	87.8	95.5
4	– 0.4	95.6	90.2	87.8	90.2	95.6
5	– 0.8	99.1	95.6	95.5	95.6	99.1

Table S10. The averaged **basic** density matrix of the horizontal layer № 5.

Number of a row in a layer	Relative coordinate ξ_2	Average density ρ; kg/m³				
		Number of the column in a layer m				
		1	2	3	4	5
		Relative coordinate ξ_1				
		– 0.8	– 0.4	0.0	0.4	0.8
1	0.8	99.4	96.2	96.0	96.2	99.4
2	0.4	96.2	91.0	88.9	91.0	96.2
3	0.0	96.0	88.9	87.5	88.9	96.0
4	– 0.4	96.2	91.0	88.9	91.0	96.2
5	– 0.8	99.4	96.2	96.0	96.2	99.4

Table S11. The averaged **basic** density matrix of the horizontal layer № 6.

**Number of a row in a layer**	**Relative coordinate ξ_2**	**Average density ρ; kg/m³**				
		**Number of the column in a layer m**				
		**1**	**2**	**3**	**4**	**5**
		**Relative coordinate ξ_1**				
		**– 0.8**	**– 0.4**	**0.0**	**0.4**	**0.8**
1	0.8	101.1	98.8	98.4	98.8	101.1
2	0.4	98.8	94.2	92.7	94.2	98.8
3	0.0	98.4	92.7	90.2	92.7	98.4
4	– 0.4	98.8	94.2	92.7	94.2	98.8
5	– 0.8	101.1	98.8	98.4	98.8	101.1

https://doi.org/10.3390/polym18060733

S4.The expanded density matrices of horizontal layers № 1 - 6

Table S12. The expanded density matrix of horizontal layer № 1 (x_1 and x_2 – coordinates, in millimetres; ξ_1 and ξ_2 – relative coordinates, and k_1 and k_2 – numbers of columns and rows).

ξ_2	x_2	k_2	Density ρ; kg/m³																				
1.00	115.0	20	107.7	107.7	107.7	107.7	107.8	107.8	107.8	107.9	107.9	108.0	108.0	108.1	108.2	108.2	108.3	108.4	108.5	108.6	108.7	108.8	108.9
0.95	109.3	19	106.9	106.9	106.9	106.9	107.0	107.0	107.1	107.1	107.2	107.3	107.4	107.5	107.6	107.7	107.8	108.0	108.1	108.3	108.4	108.6	108.8
0.90	103.5	18	106.1	106.1	106.1	106.2	106.2	106.3	106.3	106.4	106.5	106.6	106.7	106.9	107.0	107.2	107.4	107.6	107.8	108.0	108.2	108.4	108.7
0.85	97.8	17	105.3	105.4	105.4	105.4	105.5	105.6	105.6	105.7	105.9	106.0	106.2	106.3	106.5	106.7	106.9	107.2	107.4	107.7	108.0	108.3	108.6
0.80	92.0	16	104.6	104.7	104.7	104.7	104.8	104.9	105.0	105.1	105.3	105.4	105.6	105.8	106.0	106.3	106.5	106.8	107.1	107.4	107.8	108.1	108.5
0.75	86.3	15	104.0	104.0	104.0	104.1	104.2	104.3	104.4	104.5	104.7	104.9	105.1	105.3	105.6	105.9	106.1	106.5	106.8	107.2	107.6	108.0	108.4
0.70	80.5	14	103.4	103.4	103.4	103.5	103.6	103.7	103.8	104.0	104.2	104.4	104.6	104.9	105.1	105.5	105.8	106.1	106.5	106.9	107.4	107.8	108.3
0.65	74.8	13	102.8	102.8	102.8	102.9	103.0	103.1	103.3	103.5	103.7	103.9	104.1	104.4	104.7	105.1	105.5	105.8	106.3	106.7	107.2	107.7	108.2
0.60	69.0	12	102.3	102.3	102.3	102.4	102.5	102.6	102.8	103.0	103.2	103.4	103.7	104.0	104.4	104.8	105.1	105.6	106.0	106.5	107.0	107.6	108.2
0.55	63.3	11	101.8	101.8	101.8	101.9	102.0	102.2	102.3	102.5	102.8	103.0	103.3	103.7	104.0	104.4	104.9	105.3	105.8	106.3	106.9	107.5	108.1
0.50	57.5	10	101.3	101.3	101.4	101.5	101.6	101.7	101.9	102.1	102.4	102.7	103.0	103.3	103.7	104.2	104.6	105.1	105.6	106.2	106.8	107.4	108.0
0.45	51.8	9	100.9	100.9	101.0	101.1	101.2	101.3	101.5	101.8	102.0	102.3	102.7	103.0	103.4	103.9	104.4	104.9	105.4	106.0	106.6	107.3	108.0
0.40	46.0	8	100.5	100.6	100.6	100.7	100.8	101.0	101.2	101.4	101.7	102.0	102.4	102.8	103.2	103.7	104.2	104.7	105.3	105.9	106.5	107.2	107.9
0.35	40.3	7	100.2	100.2	100.3	100.4	100.5	100.7	100.9	101.2	101.4	101.8	102.1	102.5	103.0	103.5	104.0	104.5	105.1	105.7	106.4	107.1	107.9
0.30	34.5	6	99.9	100.0	100.0	100.1	100.3	100.4	100.7	100.9	101.2	101.5	101.9	102.3	102.8	103.3	103.8	104.4	105.0	105.6	106.3	107.1	107.8
0.25	28.8	5	99.7	99.7	99.8	99.9	100.0	100.2	100.4	100.7	101.0	101.3	101.7	102.2	102.6	103.1	103.7	104.3	104.9	105.6	106.3	107.0	107.8
0.20	23.0	4	99.5	99.5	99.6	99.7	99.8	100.0	100.3	100.5	100.8	101.2	101.6	102.0	102.5	103.0	103.6	104.2	104.8	105.5	106.2	107.0	107.8
0.15	17.3	3	99.4	99.4	99.4	99.6	99.7	99.9	100.1	100.4	100.7	101.1	101.5	101.9	102.4	102.9	103.5	104.1	104.7	105.4	106.2	106.9	107.7
0.10	11.5	2	99.3	99.3	99.3	99.4	99.6	99.8	100.0	100.3	100.6	101.0	101.4	101.8	102.3	102.8	103.4	104.0	104.7	105.4	106.1	106.9	107.7
0.05	5.8	1	99.2	99.2	99.3	99.4	99.5	99.7	100.0	100.2	100.6	100.9	101.3	101.8	102.3	102.8	103.4	104.0	104.7	105.4	106.1	106.9	107.7
0.00	0.0	0	99.2	99.2	99.3	99.4	99.5	99.7	99.9	100.2	100.5	100.9	101.3	101.8	102.3	102.8	103.4	104.0	104.7	105.4	106.1	106.9	107.7
		k_1	0	1	2	3	4	5	6	7	8	9	10	11	12	13	14	15	16	17	18	19	20
		x_1	0.0	5.8	11.5	17.3	23.0	28.8	34.5	40.3	46.0	51.8	57.5	63.3	69.0	74.8	80.5	86.3	92.0	97.8	103.5	109.3	115.0
		ξ_1	0.00	0.05	0.10	0.15	0.20	0.25	0.30	0.35	0.40	0.45	0.50	0.55	0.60	0.65	0.70	0.75	0.80	0.85	0.90	0.95	1.00

https://doi.org/10.3390/polym18060733

Table S13. The expanded density matrix of horizontal layer № 2 (x_1 and x_2 – coordinates, in millimetres; ξ_1 and ξ_2 – relative coordinates, and k_1 and k_2 – numbers of columns and rows).

ξ_2	x_2	k_2	Density ρ; kg/m³																				
1.00	115.0	20	103.6	103.6	103.6	103.6	103.7	103.7	103.8	103.8	103.9	104.0	104.1	104.2	104.3	104.4	104.6	104.7	104.9	105.0	105.2	105.4	105.6
0.95	109.3	19	102.5	102.5	102.5	102.5	102.6	102.6	102.7	102.8	102.9	103.1	103.2	103.3	103.5	103.7	103.9	104.1	104.3	104.6	104.8	105.1	105.4
0.90	103.5	18	101.4	101.4	101.4	101.5	101.5	101.6	101.7	101.9	102.0	102.2	102.3	102.5	102.8	103.0	103.3	103.5	103.8	104.1	104.5	104.8	105.2
0.85	97.8	17	100.4	100.4	100.4	100.5	100.6	100.7	100.8	101.0	101.1	101.3	101.5	101.8	102.1	102.3	102.7	103.0	103.4	103.7	104.1	104.6	105.0
0.80	92.0	16	99.4	99.4	99.5	99.6	99.7	99.8	99.9	100.1	100.3	100.5	100.8	101.1	101.4	101.7	102.1	102.5	102.9	103.4	103.8	104.3	104.9
0.75	86.3	15	98.5	98.6	98.6	98.7	98.8	98.9	99.1	99.3	99.5	99.8	100.1	100.4	100.8	101.1	101.6	102.0	102.5	103.0	103.5	104.1	104.7
0.70	80.5	14	97.7	97.7	97.8	97.9	98.0	98.1	98.3	98.5	98.8	99.1	99.4	99.8	100.2	100.6	101.1	101.6	102.1	102.7	103.3	103.9	104.6
0.65	74.8	13	96.9	96.9	97.0	97.1	97.2	97.4	97.6	97.8	98.1	98.4	98.8	99.2	99.6	100.1	100.6	101.1	101.7	102.3	103.0	103.7	104.4
0.60	69.0	12	96.2	96.2	96.3	96.4	96.5	96.7	96.9	97.2	97.5	97.8	98.2	98.7	99.1	99.6	100.2	100.8	101.4	102.1	102.8	103.5	104.3
0.55	63.3	11	95.5	95.6	95.6	95.7	95.9	96.1	96.3	96.6	96.9	97.3	97.7	98.2	98.7	99.2	99.8	100.4	101.1	101.8	102.5	103.3	104.2
0.50	57.5	10	94.9	95.0	95.0	95.1	95.3	95.5	95.8	96.1	96.4	96.8	97.2	97.7	98.2	98.8	99.4	100.1	100.8	101.5	102.3	103.2	104.1
0.45	51.8	9	94.4	94.4	94.5	94.6	94.8	95.0	95.3	95.6	95.9	96.3	96.8	97.3	97.8	98.4	99.1	99.8	100.5	101.3	102.2	103.1	104.0
0.40	46.0	8	93.9	93.9	94.0	94.1	94.3	94.5	94.8	95.1	95.5	95.9	96.4	96.9	97.5	98.1	98.8	99.5	100.3	101.1	102.0	102.9	103.9
0.35	40.3	7	93.5	93.5	93.6	93.7	93.9	94.1	94.4	94.7	95.1	95.6	96.1	96.6	97.2	97.8	98.5	99.3	100.1	100.9	101.9	102.8	103.8
0.30	34.5	6	93.1	93.1	93.2	93.3	93.5	93.8	94.1	94.4	94.8	95.3	95.8	96.3	96.9	97.6	98.3	99.1	99.9	100.8	101.7	102.7	103.8
0.25	28.8	5	92.8	92.8	92.9	93.0	93.2	93.5	93.8	94.1	94.5	95.0	95.5	96.1	96.7	97.4	98.1	98.9	99.8	100.7	101.6	102.6	103.7
0.20	23.0	4	92.5	92.5	92.6	92.8	93.0	93.2	93.5	93.9	94.3	94.8	95.3	95.9	96.5	97.2	98.0	98.8	99.7	100.6	101.5	102.6	103.7
0.15	17.3	3	92.3	92.3	92.4	92.6	92.8	93.0	93.3	93.7	94.1	94.6	95.1	95.7	96.4	97.1	97.9	98.7	99.6	100.5	101.5	102.5	103.6
0.10	11.5	2	92.2	92.2	92.3	92.4	92.6	92.9	93.2	93.6	94.0	94.5	95.0	95.6	96.3	97.0	97.8	98.6	99.5	100.4	101.4	102.5	103.6
0.05	5.8	1	92.1	92.1	92.2	92.3	92.5	92.8	93.1	93.5	93.9	94.4	95.0	95.6	96.2	96.9	97.7	98.6	99.4	100.4	101.4	102.5	103.6
0.00	0.0	0	92.1	92.1	92.2	92.3	92.5	92.8	93.1	93.5	93.9	94.4	94.9	95.5	96.2	96.9	97.7	98.5	99.4	100.4	101.4	102.5	103.6
		k_1	0	1	2	3	4	5	6	7	8	9	10	11	12	13	14	15	16	17	18	19	20
		x_1	0.0	5.8	11.5	17.3	23.0	28.8	34.5	40.3	46.0	51.8	57.5	63.3	69.0	74.8	80.5	86.3	92.0	97.8	103.5	109.3	115.0
		ξ_1	0.00	0.05	0.10	0.15	0.20	0.25	0.30	0.35	0.40	0.45	0.50	0.55	0.60	0.65	0.70	0.75	0.80	0.85	0.90	0.95	1.00

https://doi.org/10.3390/polym18060733

Table S14. The expanded density matrix of horizontal layer № 3 (x_1 and x_2 – coordinates, in millimetres; ξ_1 and ξ_2 – relative coordinates, and k_1 and k_2 – numbers of columns and rows).

ξ_2	x_2	k_2	**Density ρ; kg/m³**																				
1.00	115.0	20	101.0	101.0	101.0	101.0	101.1	101.1	101.2	101.2	101.3	101.4	101.5	101.7	101.8	101.9	102.1	102.3	102.4	102.6	102.8	103.0	103.3
0.95	109.3	19	99.7	99.7	99.7	99.7	99.8	99.9	100.0	100.1	100.2	100.4	100.5	100.7	100.9	101.1	101.3	101.6	101.8	102.1	102.4	102.7	103.0
0.90	103.5	18	98.4	98.4	98.5	98.5	98.6	98.7	98.8	99.0	99.1	99.3	99.5	99.8	100.0	100.3	100.6	100.9	101.2	101.6	102.0	102.4	102.8
0.85	97.8	17	97.3	97.3	97.3	97.4	97.5	97.6	97.8	97.9	98.1	98.4	98.6	98.9	99.2	99.5	99.9	100.3	100.7	101.1	101.6	102.1	102.6
0.80	92.0	16	96.2	96.2	96.2	96.3	96.4	96.6	96.7	96.9	97.2	97.4	97.7	98.1	98.4	98.8	99.2	99.7	100.2	100.7	101.2	101.8	102.4
0.75	86.3	15	95.1	95.2	95.2	95.3	95.4	95.6	95.8	96.0	96.3	96.6	96.9	97.3	97.7	98.1	98.6	99.1	99.7	100.3	100.9	101.6	102.3
0.70	80.5	14	94.2	94.2	94.3	94.4	94.5	94.7	94.9	95.1	95.4	95.8	96.2	96.6	97.0	97.5	98.1	98.6	99.2	99.9	100.6	101.3	102.1
0.65	74.8	13	93.3	93.3	93.4	93.5	93.6	93.8	94.1	94.3	94.7	95.0	95.4	95.9	96.4	96.9	97.5	98.2	98.8	99.5	100.3	101.1	101.9
0.60	69.0	12	92.4	92.5	92.5	92.7	92.8	93.0	93.3	93.6	93.9	94.3	94.8	95.3	95.8	96.4	97.0	97.7	98.4	99.2	100.0	100.9	101.8
0.55	63.3	11	91.7	91.7	91.8	91.9	92.1	92.3	92.6	92.9	93.3	93.7	94.2	94.7	95.3	95.9	96.6	97.3	98.1	98.9	99.8	100.7	101.7
0.50	57.5	10	91.0	91.0	91.1	91.2	91.4	91.6	91.9	92.3	92.7	93.1	93.6	94.2	94.8	95.4	96.2	96.9	97.7	98.6	99.5	100.5	101.5
0.45	51.8	9	90.3	90.4	90.5	90.6	90.8	91.0	91.3	91.7	92.1	92.6	93.1	93.7	94.3	95.0	95.8	96.6	97.4	98.4	99.3	100.4	101.4
0.40	46.0	8	89.8	89.8	89.9	90.0	90.2	90.5	90.8	91.2	91.6	92.1	92.7	93.3	93.9	94.7	95.4	96.3	97.2	98.1	99.1	100.2	101.3
0.35	40.3	7	89.3	89.3	89.4	89.6	89.8	90.0	90.4	90.7	91.2	91.7	92.3	92.9	93.6	94.3	95.1	96.0	96.9	97.9	99.0	100.1	101.3
0.30	34.5	6	88.9	88.9	89.0	89.1	89.3	89.6	90.0	90.4	90.8	91.3	91.9	92.6	93.3	94.1	94.9	95.8	96.7	97.8	98.8	100.0	101.2
0.25	28.8	5	88.5	88.5	88.6	88.8	89.0	89.3	89.6	90.0	90.5	91.0	91.6	92.3	93.0	93.8	94.7	95.6	96.6	97.6	98.7	99.9	101.1
0.20	23.0	4	88.2	88.2	88.3	88.5	88.7	89.0	89.3	89.8	90.2	90.8	91.4	92.1	92.8	93.6	94.5	95.4	96.4	97.5	98.6	99.8	101.1
0.15	17.3	3	88.0	88.0	88.1	88.2	88.5	88.8	89.1	89.6	90.0	90.6	91.2	91.9	92.7	93.5	94.4	95.3	96.3	97.4	98.5	99.7	101.0
0.10	11.5	2	87.8	87.8	87.9	88.1	88.3	88.6	89.0	89.4	89.9	90.5	91.1	91.8	92.5	93.4	94.3	95.2	96.2	97.3	98.5	99.7	101.0
0.05	5.8	1	87.7	87.7	87.8	88.0	88.2	88.5	88.9	89.3	89.8	90.4	91.0	91.7	92.5	93.3	94.2	95.2	96.2	97.3	98.4	99.7	101.0
0.00	0.0	0	87.7	87.7	87.8	88.0	88.2	88.5	88.9	89.3	89.8	90.3	91.0	91.7	92.4	93.3	94.2	95.1	96.2	97.3	98.4	99.7	101.0
		k_1	0	1	2	3	4	5	6	7	8	9	10	11	12	13	14	15	16	17	18	19	20
		x_1	0.0	5.8	11.5	17.3	23.0	28.8	34.5	40.3	46.0	51.8	57.5	63.3	69.0	74.8	80.5	86.3	92.0	97.8	103.5	109.3	115.0
		ξ_1	0.00	0.05	0.10	0.15	0.20	0.25	0.30	0.35	0.40	0.45	0.50	0.55	0.60	0.65	0.70	0.75	0.80	0.85	0.90	0.95	1.00

https://doi.org/10.3390/polym18060733

Table S15. The expanded density matrix of horizontal layer № 4 (x_1 and x_2 – coordinates, in millimetres; ξ_1 and ξ_2 – relative coordinates, and k_1 and k_2 – numbers of columns and rows).

ξ_2	x_2	k_2	Density ρ; kg/m³																				
1.00	115.0	20	99.9	99.9	99.9	100.0	100.0	100.0	100.1	100.2	100.3	100.3	100.4	100.6	100.7	100.8	101.0	101.1	101.3	101.5	101.6	101.8	102.0
0.95	109.3	19	98.6	98.6	98.6	98.6	98.7	98.8	98.8	99.0	99.1	99.2	99.4	99.5	99.7	99.9	100.2	100.4	100.7	100.9	101.2	101.5	101.8
0.90	103.5	18	97.3	97.3	97.3	97.4	97.4	97.5	97.7	97.8	98.0	98.1	98.4	98.6	98.8	99.1	99.4	99.7	100.1	100.4	100.8	101.2	101.6
0.85	97.8	17	96.0	96.1	96.1	96.2	96.3	96.4	96.5	96.7	96.9	97.1	97.4	97.7	98.0	98.3	98.7	99.1	99.5	100.0	100.4	100.9	101.5
0.80	92.0	16	94.9	94.9	95.0	95.0	95.1	95.3	95.5	95.7	95.9	96.2	96.5	96.8	97.2	97.6	98.0	98.5	99.0	99.5	100.1	100.7	101.3
0.75	86.3	15	93.8	93.8	93.9	94.0	94.1	94.3	94.5	94.7	95.0	95.3	95.6	96.0	96.4	96.9	97.4	97.9	98.5	99.1	99.7	100.4	101.1
0.70	80.5	14	92.8	92.8	92.9	93.0	93.1	93.3	93.5	93.8	94.1	94.4	94.8	95.3	95.7	96.2	96.8	97.4	98.0	98.7	99.4	100.2	101.0
0.65	74.8	13	91.9	91.9	91.9	92.1	92.2	92.4	92.7	93.0	93.3	93.7	94.1	94.6	95.1	95.6	96.2	96.9	97.6	98.3	99.1	99.9	100.8
0.60	69.0	12	91.0	91.0	91.1	91.2	91.4	91.6	91.9	92.2	92.5	92.9	93.4	93.9	94.5	95.1	95.7	96.4	97.2	98.0	98.8	99.7	100.7
0.55	63.3	11	90.2	90.2	90.3	90.4	90.6	90.8	91.1	91.5	91.8	92.3	92.8	93.3	93.9	94.6	95.3	96.0	96.8	97.7	98.6	99.5	100.6
0.50	57.5	10	89.4	89.5	89.6	89.7	89.9	90.1	90.4	90.8	91.2	91.7	92.2	92.8	93.4	94.1	94.8	95.6	96.5	97.4	98.4	99.4	100.4
0.45	51.8	9	88.8	88.8	88.9	89.0	89.2	89.5	89.8	90.2	90.6	91.1	91.7	92.3	92.9	93.7	94.4	95.3	96.2	97.1	98.1	99.2	100.3
0.40	46.0	8	88.2	88.2	88.3	88.5	88.7	88.9	89.3	89.7	90.1	90.6	91.2	91.8	92.5	93.3	94.1	95.0	95.9	96.9	98.0	99.1	100.3
0.35	40.3	7	87.7	87.7	87.8	87.9	88.2	88.4	88.8	89.2	89.7	90.2	90.8	91.5	92.2	93.0	93.8	94.7	95.7	96.7	97.8	99.0	100.2
0.30	34.5	6	87.2	87.2	87.3	87.5	87.7	88.0	88.4	88.8	89.3	89.8	90.4	91.1	91.9	92.7	93.5	94.5	95.5	96.5	97.7	98.8	100.1
0.25	28.8	5	86.8	86.9	87.0	87.1	87.4	87.7	88.0	88.4	88.9	89.5	90.1	90.8	91.6	92.4	93.3	94.3	95.3	96.4	97.5	98.8	100.0
0.20	23.0	4	86.5	86.5	86.7	86.8	87.1	87.4	87.7	88.2	88.7	89.2	89.9	90.6	91.4	92.2	93.1	94.1	95.1	96.3	97.4	98.7	100.0
0.15	17.3	3	86.3	86.3	86.4	86.6	86.8	87.1	87.5	87.9	88.5	89.0	89.7	90.4	91.2	92.1	93.0	94.0	95.0	96.2	97.4	98.6	100.0
0.10	11.5	2	86.1	86.1	86.2	86.4	86.7	87.0	87.3	87.8	88.3	88.9	89.6	90.3	91.1	91.9	92.9	93.9	95.0	96.1	97.3	98.6	99.9
0.05	5.8	1	86.0	86.0	86.1	86.3	86.5	86.9	87.2	87.7	88.2	88.8	89.5	90.2	91.0	91.9	92.8	93.8	94.9	96.1	97.3	98.6	99.9
0.00	0.0	0	86.0	86.0	86.1	86.3	86.5	86.8	87.2	87.7	88.2	88.8	89.4	90.2	91.0	91.9	92.8	93.8	94.9	96.0	97.3	98.6	99.9
		**k_1**	0	1	2	3	4	5	6	7	8	9	10	11	12	13	14	15	16	17	18	19	20
		**x_1**	0.0	5.8	11.5	17.3	23.0	28.8	34.5	40.3	46.0	51.8	57.5	63.3	69.0	74.8	80.5	86.3	92.0	97.8	103.5	109.3	115.0
		**ξ_1**	0.00	0.05	0.10	0.15	0.20	0.25	0.30	0.35	0.40	0.45	0.50	0.55	0.60	0.65	0.70	0.75	0.80	0.85	0.90	0.95	1.00

https://doi.org/10.3390/polym18060733

Table S16. The expanded density matrix of horizontal layer № 5 (x_1 and x_2 – coordinates, in millimetres; ξ_1 and ξ_2 – relative coordinates, and k_1 and k_2 – numbers of columns and rows).

ξ_2	x_2	k_2	Density ρ; kg/m³																				
1.00	115.0	20	100.4	100.4	100.4	100.4	100.5	100.5	100.5	100.6	100.6	100.7	100.8	100.8	100.9	101.0	101.1	101.2	101.3	101.5	101.6	101.7	101.9
0.95	109.3	19	99.1	99.1	99.1	99.1	99.2	99.2	99.3	99.4	99.5	99.6	99.7	99.9	100.0	100.2	100.4	100.6	100.8	101.0	101.2	101.5	101.7
0.90	103.5	18	97.8	97.9	97.9	97.9	98.0	98.1	98.2	98.3	98.4	98.6	98.8	99.0	99.2	99.4	99.7	100.0	100.2	100.6	100.9	101.2	101.6
0.85	97.8	17	96.7	96.7	96.7	96.8	96.9	97.0	97.1	97.3	97.4	97.6	97.9	98.1	98.4	98.7	99.0	99.4	99.7	100.1	100.6	101.0	101.5
0.80	92.0	16	95.6	95.6	95.6	95.7	95.8	95.9	96.1	96.3	96.5	96.7	97.0	97.3	97.6	98.0	98.4	98.8	99.3	99.7	100.2	100.8	101.3
0.75	86.3	15	94.5	94.5	94.6	94.7	94.8	94.9	95.1	95.3	95.6	95.9	96.2	96.6	96.9	97.4	97.8	98.3	98.8	99.4	100.0	100.6	101.2
0.70	80.5	14	93.6	93.6	93.6	93.7	93.9	94.0	94.2	94.5	94.8	95.1	95.4	95.8	96.3	96.7	97.3	97.8	98.4	99.0	99.7	100.4	101.1
0.65	74.8	13	92.6	92.7	92.7	92.8	93.0	93.2	93.4	93.7	94.0	94.3	94.7	95.2	95.7	96.2	96.8	97.4	98.0	98.7	99.4	100.2	101.0
0.60	69.0	12	91.8	91.8	91.9	92.0	92.2	92.4	92.6	92.9	93.3	93.7	94.1	94.6	95.1	95.7	96.3	96.9	97.6	98.4	99.2	100.0	100.9
0.55	63.3	11	91.0	91.1	91.1	91.3	91.4	91.6	91.9	92.2	92.6	93.0	93.5	94.0	94.6	95.2	95.8	96.6	97.3	98.1	99.0	99.9	100.8
0.50	57.5	10	90.3	90.4	90.4	90.6	90.7	91.0	91.3	91.6	92.0	92.4	92.9	93.5	94.1	94.7	95.4	96.2	97.0	97.9	98.8	99.7	100.8
0.45	51.8	9	89.7	89.7	89.8	89.9	90.1	90.4	90.7	91.0	91.5	91.9	92.4	93.0	93.7	94.3	95.1	95.9	96.7	97.6	98.6	99.6	100.7
0.40	46.0	8	89.1	89.2	89.2	89.4	89.6	89.8	90.2	90.5	91.0	91.5	92.0	92.6	93.3	94.0	94.8	95.6	96.5	97.4	98.4	99.5	100.6
0.35	40.3	7	88.6	88.6	88.7	88.9	89.1	89.4	89.7	90.1	90.5	91.0	91.6	92.2	92.9	93.7	94.5	95.3	96.3	97.3	98.3	99.4	100.6
0.30	34.5	6	88.2	88.2	88.3	88.5	88.7	89.0	89.3	89.7	90.2	90.7	91.3	91.9	92.6	93.4	94.2	95.1	96.1	97.1	98.2	99.3	100.5
0.25	28.8	5	87.8	87.8	87.9	88.1	88.3	88.6	89.0	89.4	89.8	90.4	91.0	91.6	92.4	93.2	94.0	94.9	95.9	97.0	98.1	99.2	100.5
0.20	23.0	4	87.5	87.5	87.6	87.8	88.0	88.3	88.7	89.1	89.6	90.1	90.7	91.4	92.2	93.0	93.9	94.8	95.8	96.9	98.0	99.2	100.4
0.15	17.3	3	87.3	87.3	87.4	87.6	87.8	88.1	88.5	88.9	89.4	89.9	90.6	91.3	92.0	92.8	93.7	94.7	95.7	96.8	97.9	99.1	100.4
0.10	11.5	2	87.1	87.1	87.2	87.4	87.6	87.9	88.3	88.7	89.2	89.8	90.4	91.1	91.9	92.7	93.6	94.6	95.6	96.7	97.9	99.1	100.4
0.05	5.8	1	87.0	87.0	87.1	87.3	87.5	87.8	88.2	88.6	89.2	89.7	90.4	91.1	91.8	92.7	93.6	94.5	95.6	96.7	97.9	99.1	100.4
0.00	0.0	0	87.0	87.0	87.1	87.3	87.5	87.8	88.2	88.6	89.1	89.7	90.3	91.0	91.8	92.6	93.6	94.5	95.6	96.7	97.8	99.1	100.4
		k_1	0	1	2	3	4	5	6	7	8	9	10	11	12	13	14	15	16	17	18	19	20
		x_1	0.0	5.8	11.5	17.3	23.0	28.8	34.5	40.3	46.0	51.8	57.5	63.3	69.0	74.8	80.5	86.3	92.0	97.8	103.5	109.3	115.0
		ξ_1	0.00	0.05	0.10	0.15	0.20	0.25	0.30	0.35	0.40	0.45	0.50	0.55	0.60	0.65	0.70	0.75	0.80	0.85	0.90	0.95	1.00

Table S17. The expanded density matrix of horizontal layer № 6 (x_1 and x_2 – coordinates, in millimetres; ξ_1 and ξ_2 – relative coordinates, and k_1 and k_2 – numbers of columns and rows).

ξ_2	x_2	k_2	Density ρ; kg/m³																				
1.00	115.0	20	102.4	102.4	102.4	102.4	102.4	102.4	102.4	102.5	102.5	102.5	102.5	102.5	102.5	102.6	102.6	102.6	102.7	102.7	102.7	102.8	102.8
0.95	109.3	19	101.3	101.3	101.3	101.3	101.3	101.4	101.4	101.5	101.5	101.6	101.6	101.7	101.8	101.9	102.0	102.1	102.2	102.3	102.5	102.6	102.8
0.90	103.5	18	100.2	100.2	100.2	100.2	100.3	100.3	100.4	100.5	100.6	100.7	100.8	101.0	101.1	101.3	101.4	101.6	101.8	102.0	102.2	102.5	102.7
0.85	97.8	17	99.2	99.2	99.2	99.2	99.3	99.4	99.5	99.6	99.7	99.9	100.0	100.2	100.4	100.7	100.9	101.1	101.4	101.7	102.0	102.3	102.7
0.80	92.0	16	98.2	98.2	98.2	98.3	98.4	98.5	98.6	98.7	98.9	99.1	99.3	99.5	99.8	100.1	100.4	100.7	101.0	101.4	101.8	102.2	102.7
0.75	86.3	15	97.3	97.3	97.3	97.4	97.5	97.6	97.8	97.9	98.1	98.4	98.6	98.9	99.2	99.5	99.9	100.3	100.7	101.1	101.6	102.1	102.6
0.70	80.5	14	96.4	96.5	96.5	96.6	96.7	96.8	97.0	97.2	97.4	97.7	98.0	98.3	98.7	99.0	99.5	99.9	100.4	100.9	101.4	102.0	102.6
0.65	74.8	13	95.7	95.7	95.7	95.8	95.9	96.1	96.3	96.5	96.8	97.1	97.4	97.7	98.1	98.6	99.0	99.5	100.1	100.7	101.3	101.9	102.6
0.60	69.0	12	94.9	94.9	95.0	95.1	95.2	95.4	95.6	95.9	96.1	96.5	96.8	97.2	97.7	98.1	98.7	99.2	99.8	100.4	101.1	101.8	102.5
0.55	63.3	11	94.3	94.3	94.3	94.4	94.6	94.8	95.0	95.3	95.6	95.9	96.3	96.8	97.2	97.7	98.3	98.9	99.5	100.2	101.0	101.7	102.5
0.50	57.5	10	93.6	93.7	93.7	93.8	94.0	94.2	94.4	94.7	95.1	95.4	95.9	96.3	96.8	97.4	98.0	98.6	99.3	100.0	100.8	101.6	102.5
0.45	51.8	9	93.1	93.1	93.2	93.3	93.5	93.7	93.9	94.2	94.6	95.0	95.4	95.9	96.5	97.1	97.7	98.4	99.1	99.9	100.7	101.6	102.5
0.40	46.0	8	92.6	92.6	92.7	92.8	93.0	93.2	93.5	93.8	94.2	94.6	95.1	95.6	96.1	96.8	97.4	98.1	98.9	99.7	100.6	101.5	102.5
0.35	40.3	7	92.1	92.2	92.2	92.4	92.6	92.8	93.1	93.4	93.8	94.2	94.7	95.3	95.9	96.5	97.2	97.9	98.7	99.6	100.5	101.5	102.5
0.30	34.5	6	91.8	91.8	91.9	92.0	92.2	92.4	92.7	93.1	93.5	93.9	94.4	95.0	95.6	96.3	97.0	97.8	98.6	99.5	100.4	101.4	102.4
0.25	28.8	5	91.4	91.5	91.6	91.7	91.9	92.1	92.4	92.8	93.2	93.7	94.2	94.8	95.4	96.1	96.8	97.6	98.5	99.4	100.3	101.4	102.4
0.20	23.0	4	91.2	91.2	91.3	91.4	91.6	91.9	92.2	92.6	93.0	93.5	94.0	94.6	95.2	95.9	96.7	97.5	98.4	99.3	100.3	101.3	102.4
0.15	17.3	3	91.0	91.0	91.1	91.2	91.4	91.7	92.0	92.4	92.8	93.3	93.8	94.4	95.1	95.8	96.6	97.4	98.3	99.2	100.2	101.3	102.4
0.10	11.5	2	90.8	90.9	90.9	91.1	91.3	91.6	91.9	92.2	92.7	93.2	93.7	94.3	95.0	95.7	96.5	97.3	98.2	99.2	100.2	101.3	102.4
0.05	5.8	1	90.7	90.8	90.9	91.0	91.2	91.5	91.8	92.2	92.6	93.1	93.7	94.3	94.9	95.7	96.5	97.3	98.2	99.2	100.2	101.3	102.4
0.00	0.0	0	90.7	90.7	90.8	91.0	91.2	91.4	91.8	92.1	92.6	93.1	93.6	94.3	94.9	95.7	96.4	97.3	98.2	99.2	100.2	101.3	102.4
		**k_1**	0	1	2	3	4	5	6	7	8	9	10	11	12	13	14	15	16	17	18	19	20
		**x_1**	0.0	5.8	11.5	17.3	23.0	28.8	34.5	40.3	46.0	51.8	57.5	63.3	69.0	74.8	80.5	86.3	92.0	97.8	103.5	109.3	115.0
		**ξ_1**	0.00	0.05	0.10	0.15	0.20	0.25	0.30	0.35	0.40	0.45	0.50	0.55	0.60	0.65	0.70	0.75	0.80	0.85	0.90	0.95	1.00

https://doi.org/10.3390/polym18060733

S5.Density in the vertical perpendicular section

Table S18. The basic density matrix in vertical perpendicular section (x_1 and x_3 – coordinates, in millimetres; ξ_1 and ξ_3 – relative coordinates, and n_1 and n_3 – numbers of columns and rows).

ξ_3	x_3	n_3	Density ρ; kg/m³																				
0.92	91.9	6	90.7	90.7	90.8	91.0	91.2	91.4	91.8	92.1	92.6	93.1	93.6	94.3	94.9	95.7	96.4	97.3	98.2	99.2	100.2	101.3	102.4
0.75	75.2	5	87.0	87.0	87.1	87.3	87.5	87.8	88.2	88.6	89.1	89.7	90.3	91.0	91.8	92.6	93.6	94.5	95.6	96.7	97.8	99.1	100.4
0.58	58.5	4	86.0	86.0	86.1	86.3	86.5	86.8	87.2	87.7	88.2	88.8	89.4	90.2	91.0	91.9	92.8	93.8	94.9	96.0	97.3	98.6	99.9
0.42	41.8	3	87.7	87.7	87.8	88.0	88.2	88.5	88.9	89.3	89.8	90.3	91.0	91.7	92.4	93.3	94.2	95.1	96.2	97.3	98.4	99.7	101.0
0.25	25.1	2	92.1	92.1	92.2	92.3	92.5	92.8	93.1	93.5	93.9	94.4	94.9	95.5	96.2	96.9	97.7	98.5	99.4	100.4	101.4	102.5	103.6
0.08	8.4	1	99.2	99.2	99.3	99.4	99.5	99.7	99.9	100.2	100.5	100.9	101.3	101.8	102.3	102.8	103.4	104.0	104.7	105.4	106.1	106.9	107.7
		n_1	0	1	2	3	4	5	6	7	8	9	10	11	12	13	14	15	16	17	18	19	20
		x_1	0.0	5.8	11.5	17.3	23.0	28.8	34.5	40.3	46.0	51.8	57.5	63.3	69.0	74.8	80.5	86.3	92.0	97.8	103.5	109.3	115.0
		ξ_1	0.00	0.05	0.10	0.15	0.20	0.25	0.30	0.35	0.40	0.45	0.50	0.55	0.60	0.65	0.70	0.75	0.80	0.85	0.90	0.95	1.00

Table S19. The expanded density matrix in vertical perpendicular section (x_1 and x_3 – coordinates, in millimetres; ξ_1 and ξ_3 – relative coordinates, and n_1 and n_3 – numbers of columns and rows).

ξ_3	x_3	n_3	Density ρ; kg/m³																				
1.00	100.0	18	90.7	90.7	90.8	91.0	91.2	91.4	91.8	92.1	92.6	93.1	93.6	94.3	94.9	95.7	96.4	97.3	98.2	99.2	100.2	101.3	102.4
0.94	94.4	17	89.2	89.2	89.3	89.4	89.7	89.9	90.3	90.7	91.2	91.7	92.3	92.9	93.6	94.4	95.2	96.1	97.1	98.1	99.2	100.4	101.6
0.89	88.9	16	87.9	88.0	88.1	88.2	88.4	88.7	89.1	89.5	90.0	90.5	91.2	91.8	92.6	93.4	94.3	95.2	96.2	97.3	98.4	99.6	100.9
0.83	83.3	15	87.0	87.0	87.1	87.3	87.5	87.8	88.2	88.6	89.1	89.7	90.3	91.0	91.8	92.6	93.5	94.5	95.6	96.7	97.8	99.1	100.4
0.78	77.8	14	86.3	86.4	86.5	86.6	86.9	87.2	87.6	88.0	88.5	89.1	89.8	90.5	91.3	92.1	93.1	94.1	95.1	96.3	97.5	98.7	100.1
0.72	72.2	13	86.0	86.0	86.1	86.3	86.6	86.9	87.2	87.7	88.2	88.8	89.5	90.2	91.0	91.9	92.8	93.8	94.9	96.0	97.3	98.5	99.9
0.67	66.7	12	86.0	86.0	86.1	86.3	86.5	86.8	87.2	87.7	88.2	88.8	89.5	90.2	91.0	91.8	92.8	93.8	94.9	96.0	97.3	98.5	99.9
0.61	61.1	11	86.2	86.3	86.4	86.5	86.8	87.1	87.5	87.9	88.4	89.0	89.7	90.4	91.2	92.1	93.0	94.0	95.1	96.2	97.5	98.7	100.1
0.56	55.6	10	86.8	86.8	86.9	87.1	87.3	87.6	88.0	88.5	89.0	89.6	90.2	90.9	91.7	92.6	93.5	94.5	95.5	96.7	97.9	99.1	100.5
0.50	50.0	9	87.6	87.7	87.8	87.9	88.2	88.5	88.8	89.3	89.8	90.3	91.0	91.7	92.4	93.3	94.2	95.1	96.2	97.3	98.4	99.7	101.0
0.44	44.4	8	88.8	88.9	88.9	89.1	89.3	89.6	90.0	90.4	90.9	91.4	92.0	92.7	93.4	94.2	95.1	96.0	97.0	98.1	99.2	100.4	101.7
0.39	38.9	7	90.3	90.3	90.4	90.6	90.8	91.1	91.4	91.8	92.2	92.8	93.4	94.0	94.7	95.5	96.3	97.2	98.1	99.1	100.2	101.3	102.5
0.33	33.3	6	92.1	92.1	92.2	92.3	92.5	92.8	93.1	93.5	93.9	94.4	94.9	95.5	96.2	96.9	97.7	98.5	99.4	100.4	101.4	102.5	103.6
0.28	27.8	5	94.1	94.2	94.2	94.4	94.6	94.8	95.1	95.4	95.8	96.3	96.8	97.4	98.0	98.6	99.4	100.1	101.0	101.8	102.8	103.7	104.8

https://doi.org/10.3390/polym18060733

0.22	22.2	4	96.5	96.5	96.6	96.7	96.9	97.1	97.4	97.7	98.0	98.5	98.9	99.4	100.0	100.6	101.2	101.9	102.7	103.5	104.3	105.2	106.2
0.17	16.7	3	99.2	99.2	99.3	99.4	99.5	99.7	99.9	100.2	100.5	100.9	101.3	101.8	102.3	102.8	103.4	104.0	104.6	105.4	106.1	106.9	107.7
0.11	11.1	2	102.1	102.2	102.2	102.3	102.4	102.6	102.8	103.0	103.3	103.6	104.0	104.4	104.8	105.2	105.7	106.3	106.8	107.4	108.1	108.7	109.4
0.06	5.6	1	105.4	105.4	105.5	105.6	105.7	105.8	106.0	106.1	106.4	106.6	106.9	107.2	107.6	107.9	108.3	108.8	109.2	109.7	110.2	110.8	111.3
0.00	0.0	0	109.0	109.0	109.1	109.1	109.2	109.3	109.4	109.5	109.7	109.9	110.1	110.3	110.6	110.9	111.2	111.5	111.8	112.2	112.6	113.0	113.4
		**n_1**	0	1	2	3	4	5	6	7	8	9	10	11	12	13	14	15	16	17	18	19	20
		**x_1**	0.0	5.8	11.5	17.3	23.0	28.8	34.5	40.3	46.0	51.8	57.5	63.3	69.0	74.8	80.5	86.3	92.0	97.8	103.5	109.3	115.0
		**ξ_1**	0.00	0.05	0.10	0.15	0.20	0.25	0.30	0.35	0.40	0.45	0.50	0.55	0.60	0.65	0.70	0.75	0.80	0.85	0.90	0.95	1.00

S6.Density in the vertical diagonal section

Table S20. The basic density matrix in vertical diagonal section (x_1, x_3, and x_d – coordinates, in millimetres; ξ_1, ξ_3, and ξ_d – relative coordinates, and n_d and n_3 – numbers of columns and rows).

**ξ_3**	**x_3**	**n_3**	**Density ρ; kg/m³**																				
0.92	91.9	6	90.7	90.8	90.9	91.2	91.6	92.1	92.7	93.4	94.2	95.0	95.9	96.8	97.7	98.6	99.5	100.3	101.0	101.7	102.2	102.6	102.8
0.75	75.2	5	87.0	87.0	87.2	87.6	88.0	88.6	89.3	90.1	91.0	91.9	92.9	94.0	95.1	96.2	97.3	98.3	99.3	100.1	100.9	101.5	101.9
0.58	58.5	4	86.0	86.0	86.2	86.6	87.1	87.7	88.4	89.2	90.1	91.1	92.2	93.3	94.5	95.6	96.8	97.9	99.0	100.0	100.8	101.5	102.0
0.42	41.8	3	87.7	87.7	87.9	88.2	88.7	89.3	90.0	90.7	91.6	92.6	93.6	94.7	95.8	96.9	98.1	99.1	100.2	101.1	102.0	102.7	103.3
0.25	25.1	2	92.1	92.1	92.3	92.6	93.0	93.5	94.1	94.7	95.5	96.3	97.2	98.2	99.1	100.1	101.1	102.0	102.9	103.7	104.5	105.1	105.6
0.08	8.4	1	99.2	99.2	99.3	99.6	99.8	100.2	100.7	101.2	101.7	102.3	103.0	103.7	104.4	105.1	105.8	106.5	107.1	107.7	108.2	108.6	108.9
		**n_1**	0	1	2	3	4	5	6	7	8	9	10	11	12	13	14	15	16	17	18	19	20
		**x_1**	0.0	5.8	11.5	17.3	23.0	28.8	34.5	40.3	46.0	51.8	57.5	63.3	69.0	74.8	80.5	86.3	92.0	97.8	103.5	109.3	115.0
		**x_d**	0.0	8.1	16.3	24.4	32.5	40.7	48.8	56.9	65.1	73.2	81.3	89.4	97.6	105.7	113.8	122.0	130.1	138.2	146.4	154.5	162.6
		**ξ_d**	0.00	0.05	0.10	0.15	0.20	0.25	0.30	0.35	0.40	0.45	0.50	0.55	0.60	0.65	0.70	0.75	0.80	0.85	0.90	0.95	1.00

https://doi.org/10.3390/polym18060733

Table S21. The basic density matrix for vertical diagonal section, columns № 0 – 14, at $\Delta x_d = \Delta x_1 = \Delta x_2$ = 5.8 mm (x_3 and x_d – coordinates, in millimetres; ξ_3 and ξ_d – relative coordinates, and n_d and n_3 – numbers of columns and rows).

ξ_3	x_3	n_3	Density ρ; kg/m³														
0.92	91.9	6	90.8	90.8	90.8	90.9	91.1	91.3	91.7	92.0	92.5	92.9	93.4	94.0	94.6	95.2	95.8
0.75	75.2	5	87.1	87.0	87.1	87.2	87.4	87.7	88.1	88.5	89.0	89.5	90.1	90.8	91.4	92.1	92.8
0.58	58.5	4	86.1	86.0	86.1	86.2	86.4	86.7	87.1	87.6	88.1	88.7	89.3	90.0	90.7	91.5	92.3
0.42	41.8	3	87.8	87.7	87.8	87.9	88.1	88.4	88.7	89.1	89.6	90.2	90.7	91.4	92.0	92.7	93.4
0.25	25.1	2	92.2	92.1	92.1	92.3	92.4	92.7	93.0	93.4	93.8	94.3	94.8	95.4	96.0	96.6	97.2
0.08	8.4	1	99.3	99.2	99.2	99.3	99.5	99.7	99.9	100.2	100.5	100.9	101.3	101.7	102.2	102.7	103.2
		n_d	0	1	2	3	4	5	6	7	8	9	10	11	12	13	14
		x_d	0.0	5.8	11.5	17.3	23.0	28.8	34.5	40.3	46.0	51.8	57.5	63.3	69.0	74.8	80.5
		ξ_d	0.0	0.0	0.1	0.1	0.1	0.2	0.2	0.2	0.3	0.3	0.4	0.4	0.4	0.5	0.5

Table S21 (continuation). The basic density matrix for vertical diagonal section, columns № 15 – 28, at $\Delta x_d = \Delta x_1 = \Delta x_2$ = 5.8 mm (x_3 and x_d – coordinates, in millimetres; ξ_3 and ξ_d – relative coordinates, and n_d and n_3 – numbers of columns and rows).

ξ_3	x_3	n_3	Density ρ; kg/m³													
0.92	91.9	6	96.4	97.1	97.7	98.4	99.0	99.6	100.2	100.8	101.3	101.8	102.2	102.6	103.0	103.3
0.75	75.2	5	93.5	94.2	94.9	95.6	96.2	96.9	97.5	98.1	98.6	99.1	99.5	99.9	100.2	100.4
0.58	58.5	4	93.1	94.0	94.8	95.7	96.5	97.4	98.3	99.1	99.9	100.7	101.5	102.2	102.9	103.6
0.42	41.8	3	94.2	94.9	95.7	96.4	97.2	97.9	98.7	99.4	100.1	100.7	101.3	101.9	102.4	102.9
0.25	25.1	2	97.9	98.6	99.3	100.0	100.8	101.5	102.2	102.9	103.6	104.2	104.9	105.5	106.1	106.6
0.08	8.4	1	103.7	104.3	104.8	105.4	106.0	106.5	107.1	107.6	108.2	108.7	109.3	109.8	110.2	110.7
		n_d	15	16	17	18	19	20	21	22	23	24	25	26	27	28
		x_d	86.3	92.0	97.8	103.5	109.3	115.0	120.8	126.5	132.3	138.0	143.8	149.5	155.3	161.0
		ξ_d	0.5	0.6	0.6	0.6	0.7	0.7	0.7	0.8	0.8	0.8	0.9	0.9	1.0	1.0

https://doi.org/10.3390/polym18060733

Table S22. The expanded density matrix in vertical diagonal section, columns № 0 - 14 (x_3 and x_d – coordinates, in millimetres; ξ_3 and ξ_d – relative coordinates, and n_d and n_3 – numbers of columns and rows).

ξ_3	x_3	n_3	Density ρ; kg/m³														
1.00	100.0	18	91.1	91.0	91.1	91.1	91.0	91.7	91.6	91.6	92.7	93.0	93.4	93.9	94.5	95.2	95.9
0.94	94.4	17	89.6	89.5	89.6	89.6	89.5	90.1	90.1	90.1	91.2	91.6	92.0	92.6	93.2	93.9	94.6
0.89	88.9	16	88.3	88.2	88.3	88.3	88.2	88.9	89.0	89.0	90.1	90.4	90.9	91.5	92.1	92.9	93.7
0.83	83.3	15	87.3	87.2	87.3	87.3	87.3	87.9	88.2	88.2	89.2	89.6	90.1	90.7	91.4	92.1	92.9
0.78	77.8	14	86.6	86.6	86.6	86.6	86.7	87.3	87.6	87.6	88.5	89.0	89.5	90.1	90.8	91.6	92.4
0.72	72.2	13	86.3	86.2	86.3	86.3	86.4	86.9	87.3	87.3	88.2	88.7	89.2	89.9	90.6	91.3	92.1
0.67	66.7	12	86.2	86.1	86.2	86.2	86.4	86.9	87.3	87.3	88.2	88.7	89.2	89.9	90.6	91.3	92.1
0.61	61.1	11	86.5	86.4	86.5	86.5	86.6	87.1	87.6	87.6	88.4	88.9	89.5	90.1	90.8	91.6	92.4
0.56	55.6	10	87.0	86.9	87.0	87.0	87.2	87.6	88.2	88.2	88.9	89.4	90.0	90.6	91.3	92.1	92.9
0.50	50.0	9	87.8	87.8	87.8	87.8	88.1	88.5	89.1	89.1	89.7	90.2	90.8	91.4	92.1	92.8	93.6
0.44	44.4	8	89.0	88.9	89.0	89.0	89.2	89.6	90.2	90.2	90.8	91.3	91.9	92.5	93.1	93.8	94.6
0.39	38.9	7	90.4	90.4	90.4	90.4	90.7	91.0	91.6	91.6	92.2	92.7	93.2	93.8	94.4	95.1	95.8
0.33	33.3	6	92.2	92.1	92.2	92.2	92.4	92.7	93.3	93.3	93.8	94.3	94.8	95.4	96.0	96.6	97.3
0.28	27.8	5	94.2	94.2	94.2	94.2	94.5	94.7	95.3	95.3	95.8	96.2	96.7	97.2	97.8	98.4	99.0
0.22	22.2	4	96.6	96.6	96.6	96.6	96.8	97.1	97.6	97.6	98.0	98.4	98.8	99.3	99.8	100.4	101.0
0.17	16.7	3	99.3	99.2	99.3	99.3	99.5	99.7	100.2	100.2	100.5	100.9	101.3	101.7	102.2	102.6	103.2
0.11	11.1	2	102.2	102.2	102.2	102.2	102.4	102.6	103.0	103.0	103.3	103.6	104.0	104.3	104.8	105.2	105.6
0.06	5.6	1	105.5	105.5	105.5	105.5	105.6	105.8	106.1	106.1	106.4	106.6	106.9	107.3	107.6	107.9	108.3
0.00	0.0	0	109.1	109.0	109.0	109.0	109.2	109.3	109.6	109.6	109.7	110.0	110.2	110.4	110.7	111.0	111.3
		n_d	0	1	2	3	4	5	6	7	8	9	10	11	12	13	14
		x_d	0.0	5.8	11.5	17.3	23.0	28.8	34.5	40.3	46.0	51.8	57.5	63.3	69.0	74.8	80.5
		ξ_d	0.0	0.0	0.1	0.1	0.1	0.2	0.2	0.2	0.3	0.3	0.4	0.4	0.4	0.5	0.5

https://doi.org/10.3390/polym18060733

Table S22 (continuation). The expanded density matrix in vertical diagonal section, columns № 15 - 28 (x_3, and x_d – coordinates, in millimetres; ξ_3 and ξ_d – relative coordinates, and n_d and n_3 – numbers of columns and rows).).

ξ_3	x_3	n_3	**Density ρ; kg/m³**													
1.00	100.0	18	96.7	97.5	97.3	98.1	99.0	99.8	100.6	100.3	101.0	101.6	102.1	102.6	102.9	103.1
0.94	94.4	17	95.4	96.3	96.2	97.1	98.0	98.8	99.6	99.5	100.2	100.9	101.4	101.9	102.3	102.5
0.89	88.9	16	94.5	95.3	95.4	96.3	97.2	98.0	98.9	98.9	99.6	100.3	100.9	101.4	101.8	102.2
0.83	83.3	15	93.7	94.6	94.8	95.7	96.6	97.5	98.3	98.4	99.2	99.9	100.6	101.1	101.6	101.9
0.78	77.8	14	93.2	94.1	94.4	95.3	96.2	97.1	98.0	98.2	99.0	99.7	100.4	100.9	101.4	101.9
0.72	72.2	13	93.0	93.9	94.2	95.1	96.0	96.9	97.8	98.1	98.9	99.7	100.3	100.9	101.5	101.9
0.67	66.7	12	93.0	93.8	94.3	95.2	96.1	97.0	97.8	98.2	99.0	99.8	100.5	101.1	101.7	102.1
0.61	61.1	11	93.2	94.1	94.6	95.5	96.3	97.2	98.1	98.5	99.3	100.1	100.8	101.4	102.0	102.5
0.56	55.6	10	93.7	94.5	95.1	95.9	96.8	97.7	98.5	99.0	99.8	100.6	101.3	101.9	102.5	103.0
0.50	50.0	9	94.4	95.2	95.8	96.6	97.5	98.3	99.1	99.7	100.5	101.2	101.9	102.6	103.1	103.7
0.44	44.4	8	95.3	96.1	96.7	97.5	98.4	99.2	100.0	100.6	101.3	102.0	102.7	103.4	103.9	104.5
0.39	38.9	7	96.5	97.3	97.9	98.7	99.5	100.2	101.0	101.6	102.3	103.0	103.7	104.3	104.9	105.4
0.33	33.3	6	98.0	98.7	99.3	100.0	100.8	101.5	102.2	102.8	103.5	104.2	104.8	105.4	106.0	106.5
0.28	27.8	5	99.6	100.3	100.9	101.6	102.3	103.0	103.6	104.2	104.9	105.5	106.1	106.7	107.3	107.8
0.22	22.2	4	101.5	102.1	102.7	103.4	104.0	104.6	105.3	105.8	106.4	107.0	107.6	108.2	108.7	109.2
0.17	16.7	3	103.7	104.2	104.8	105.3	105.9	106.5	107.1	107.6	108.2	108.7	109.3	109.8	110.3	110.7
0.11	11.1	2	106.1	106.6	107.1	107.6	108.1	108.6	109.1	109.6	110.1	110.6	111.1	111.5	112.0	112.4
0.06	5.6	1	108.7	109.1	109.5	110.0	110.4	110.9	111.3	111.7	112.2	112.6	113.0	113.5	113.9	114.3
0.00	0.0	0	111.6	111.9	112.3	112.6	113.0	113.3	113.7	114.1	114.5	114.8	115.2	115.6	115.9	116.3
		n_d	15	16	17	18	19	20	21	22	23	24	25	26	27	28
		x_d	86.3	92.0	97.8	103.5	109.3	115.0	120.8	126.5	132.3	138.0	143.8	149.5	155.3	161.0
		ξ_d	0.5	0.6	0.6	0.6	0.7	0.7	0.7	0.8	0.8	0.8	0.9	0.9	1.0	1.0

https://doi.org/10.3390/polym18060733

S7.Charts of density distribution in the horizontal layers № 1 – 6

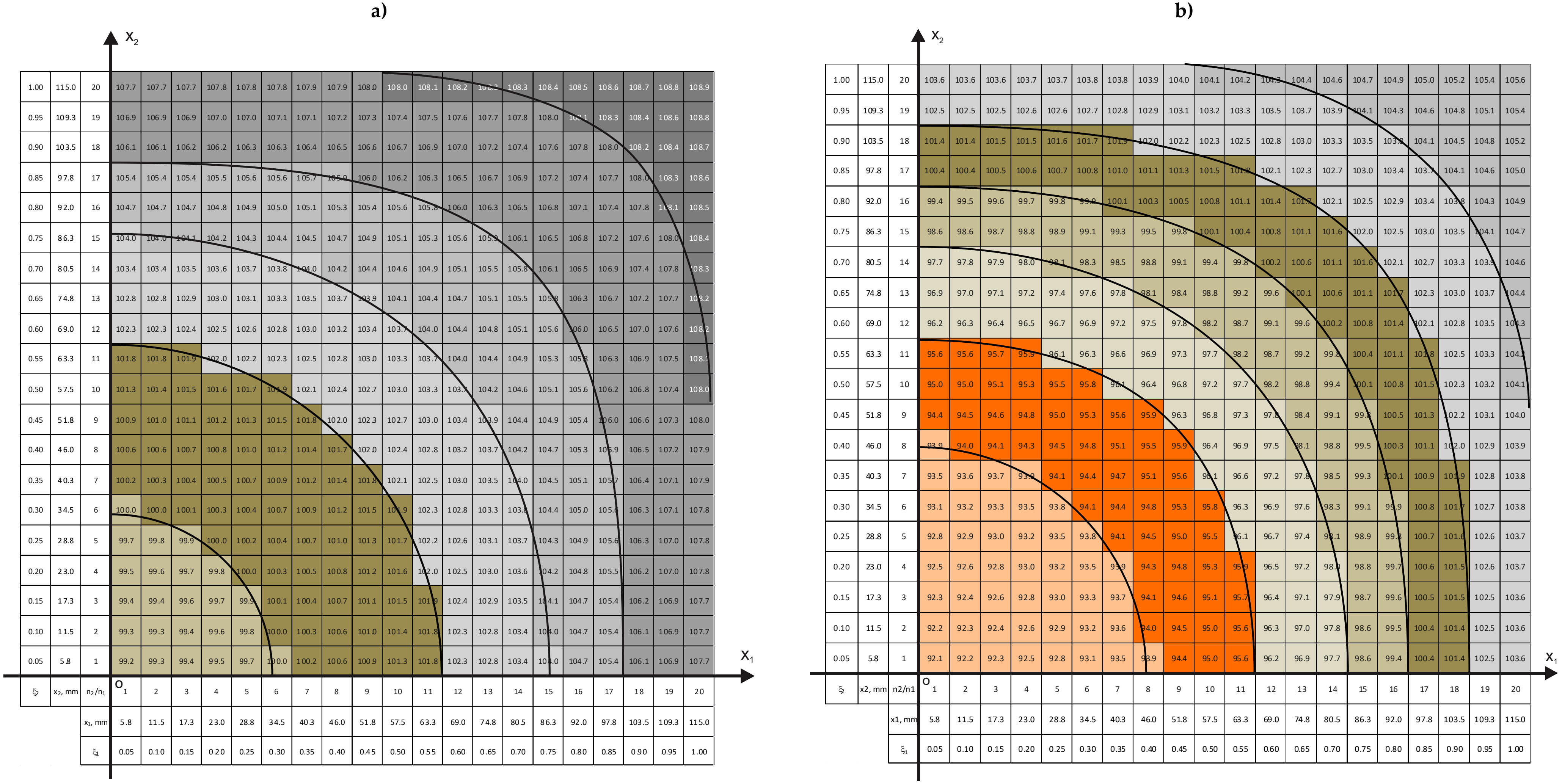

Figure S1. Charts of density distribution in the horizontal layers **a)** № 1 and **b)** № 2; parallel to the plane x_1ox_2.

https://doi.org/10.3390/polym18060733

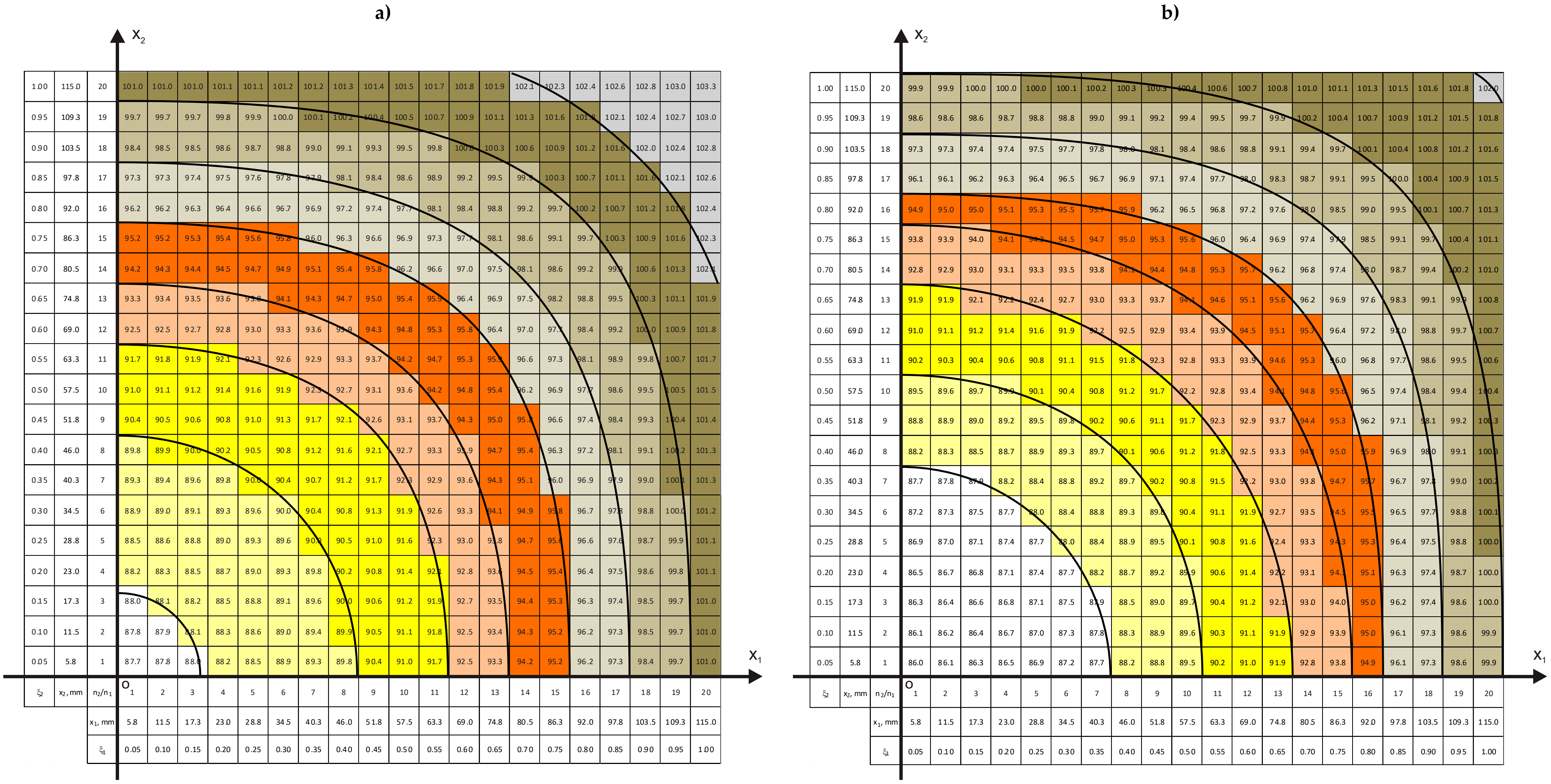

Figure S2. Charts of density distribution in the horizontal layers **a)** № 3 and **b)** № 4; parallel to the plane x_1ox_2.

https://doi.org/10.3390/polym18060733

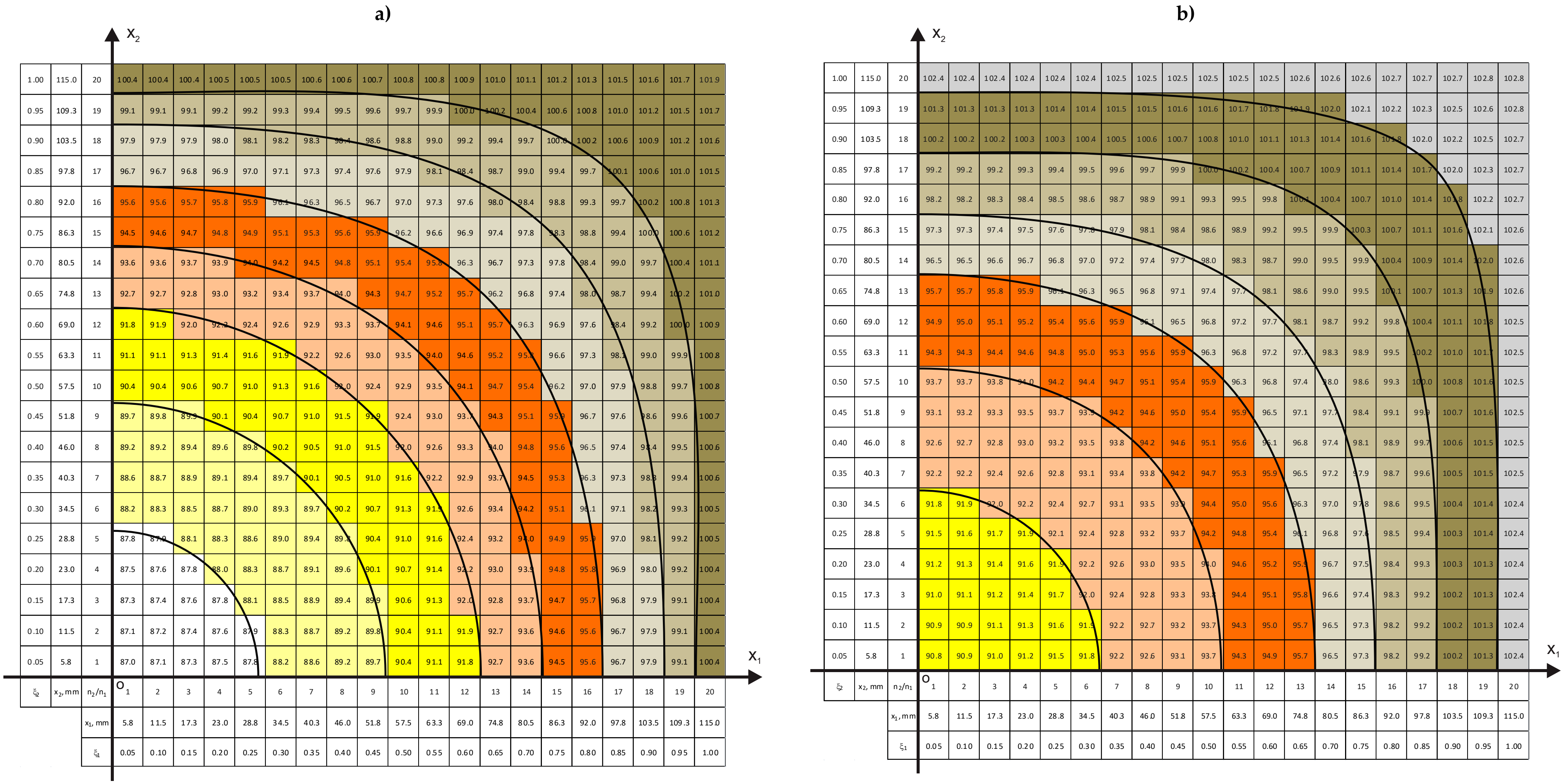

Figure S3. Charts of density distribution in the horizontal layers **a)** № 5 and **b)** № 6; parallel to the plane x_1ox_2.

https://doi.org/10.3390/polym18060733

S8.Graphs of density and gradients in the horizontal layers № 1 - 6

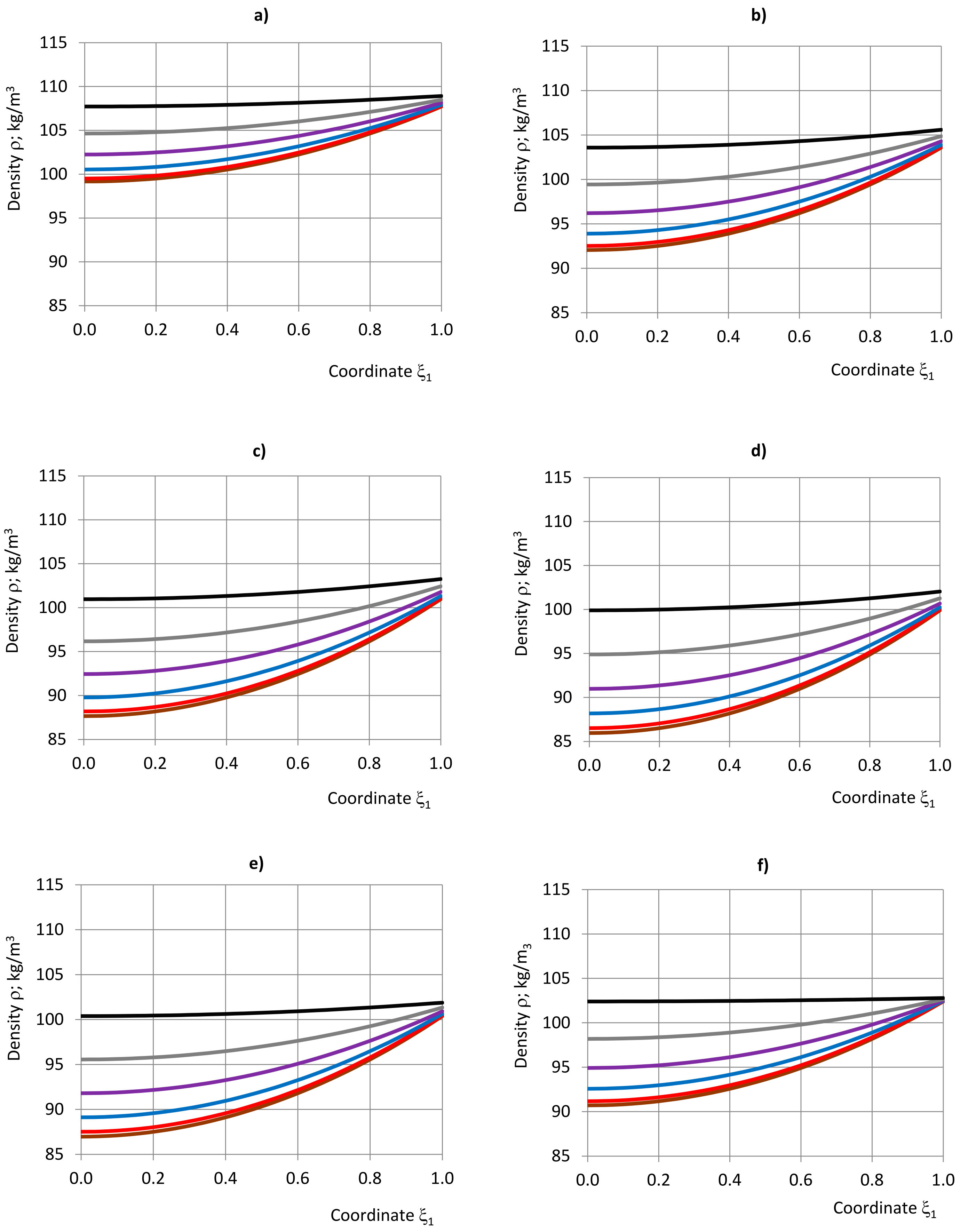

https://doi.org/10.3390/polym18060733

Figure S4. Density depending on coordinate ξ₁ at fixed values of coordinate ξ₂ 1) $\xi_2 = 0.0$ (Black), 2) $\xi_2 = 0.2$ (Grey), 3) $\xi_2 = 0.4$ (Violet), 4) $\xi_2 = 0.6$ (Blue), 5) $\xi_2 = 0.8$ (Red), and 6) $\xi_2 = 1.0$ (Brown); **a) – f)** horizontal layers № 1 – 6.

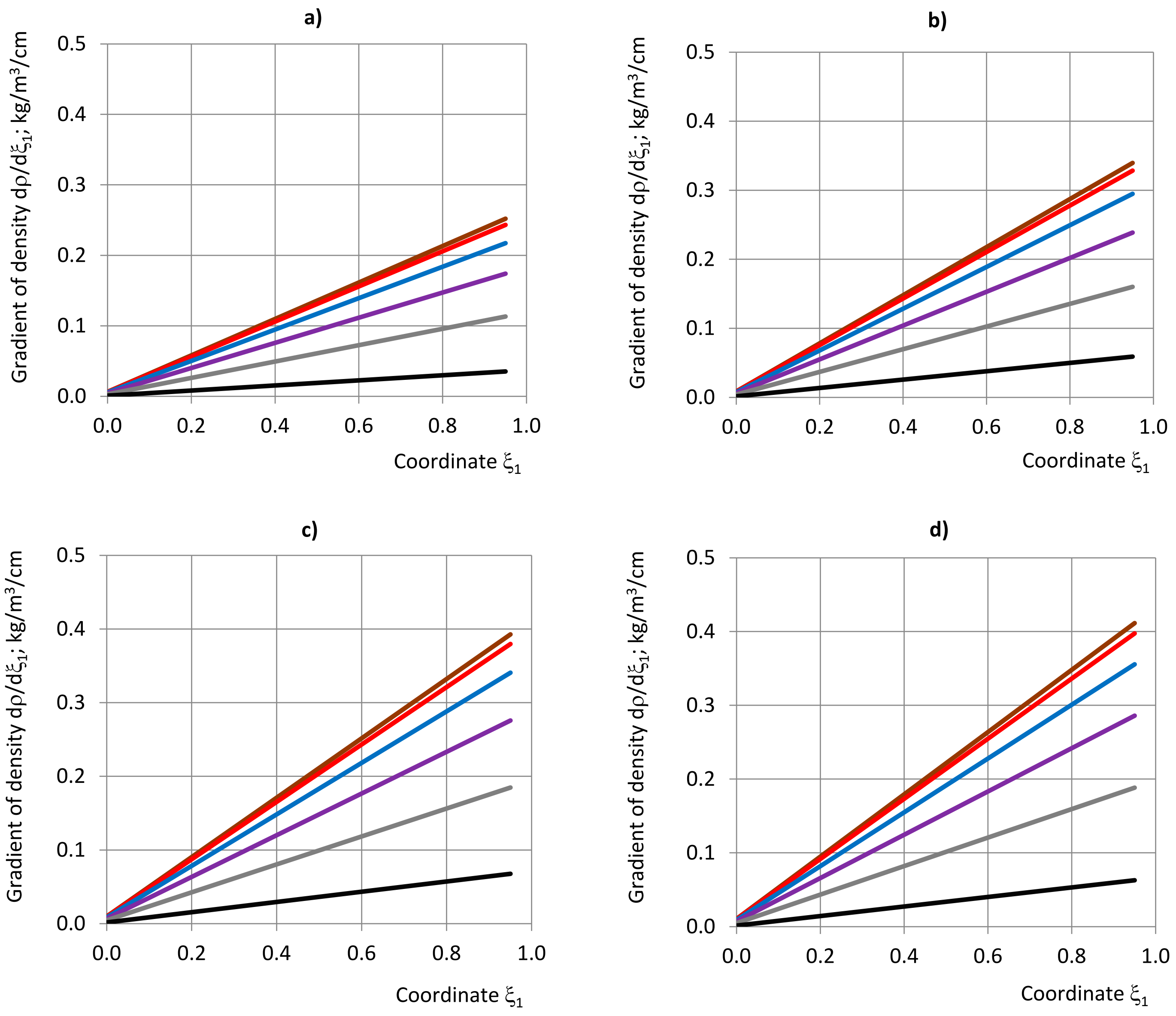

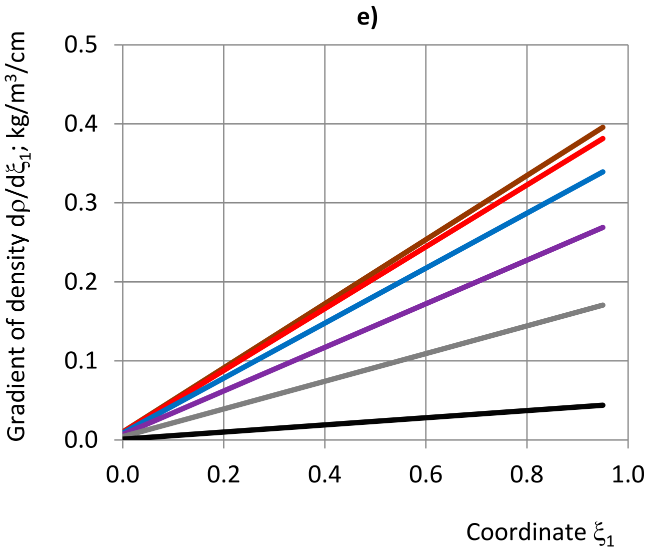

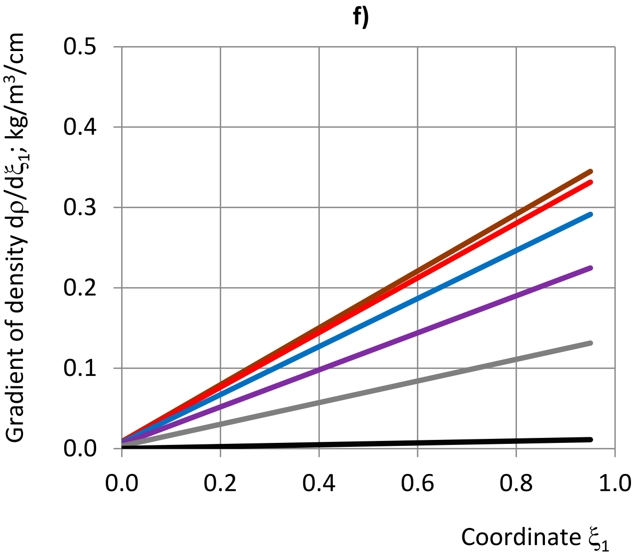

Figure S5. Density gradient depending on coordinate ξ_1 at fixed values of coordinate ξ_2 1) $\xi_2 = 0.0$ (Black), 2) $\xi_2 = 0.2$ (Grey), 3) $\xi_2 = 0.4$ (Violet), 4) $\xi_2 = 0.6$ (Blue), 5) $\xi_2 = 0.8$ (Red), and 6) $\xi_2 = 0.95$ (Brown); **a) – f)** horizontal layers № 1 – 6.

S9.Graphs of density and gradients in the vertical perpendicular section

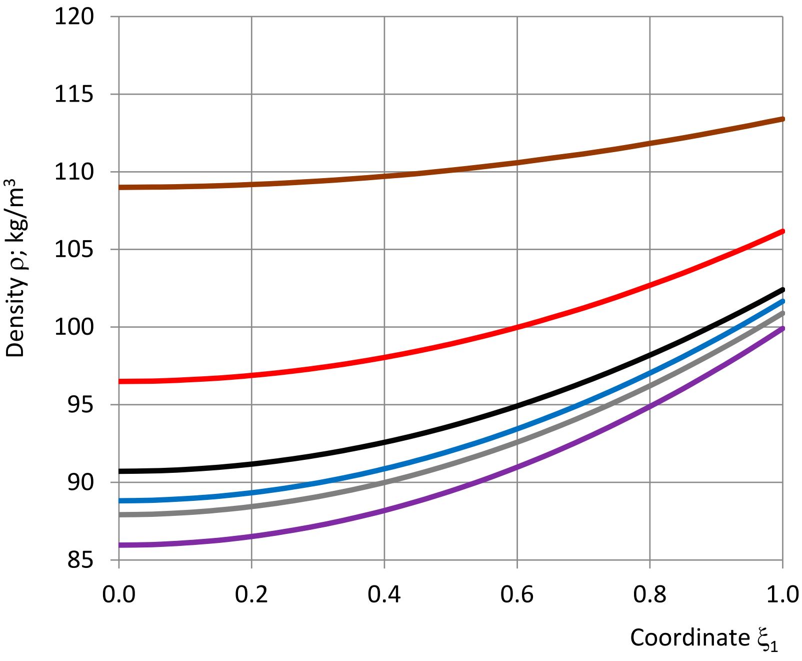

Figure S6. Density in the vertical perpendicular section depending on coordinate ξ_1 at fixed values of coordinate ξ_3 1) $\xi_3 = 0.00$ (Black), 2) $\xi_3 = 0.22$ (Grey), 3) $\xi_3 = 0.44$ (Violet), 4) $\xi_3 = 0.67$ (Blue), 5) $\xi_3 = 0.89$ (Red), and 6) $\xi_3 = 1.00$ (Brown).

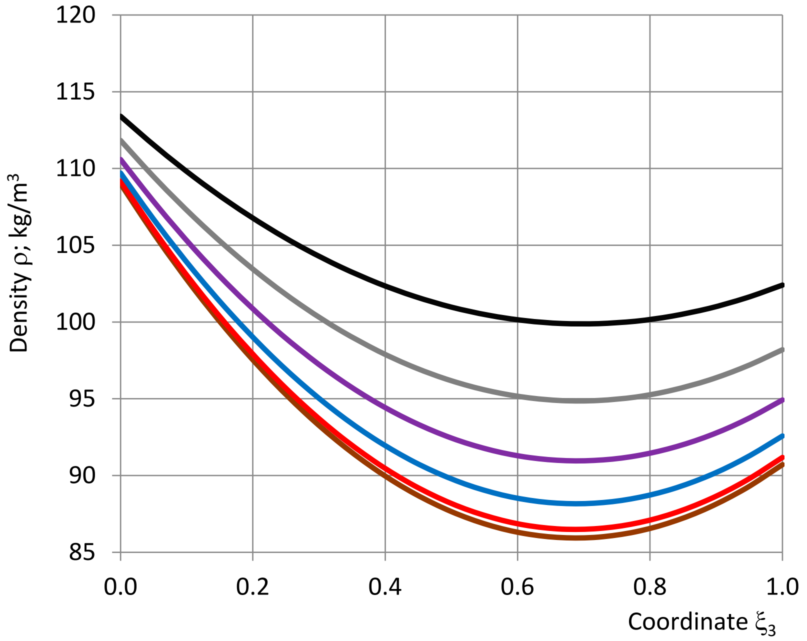

Figure S7. Density in the vertical perpendicular section depending on coordinate ξ_3 at fixed values of coordinate ξ_1 1) $\xi_1 = 0.0$ (Black), 2) $\xi_1 = 0.2$ (Grey), 3) $\xi_1 = 0.4$ (Violet), 4) $\xi_1 = 0.6$ (Blue), 5) $\xi_1 = 0.8$ (Red), and 6) $\xi_1 = 1.00$ (Brown).

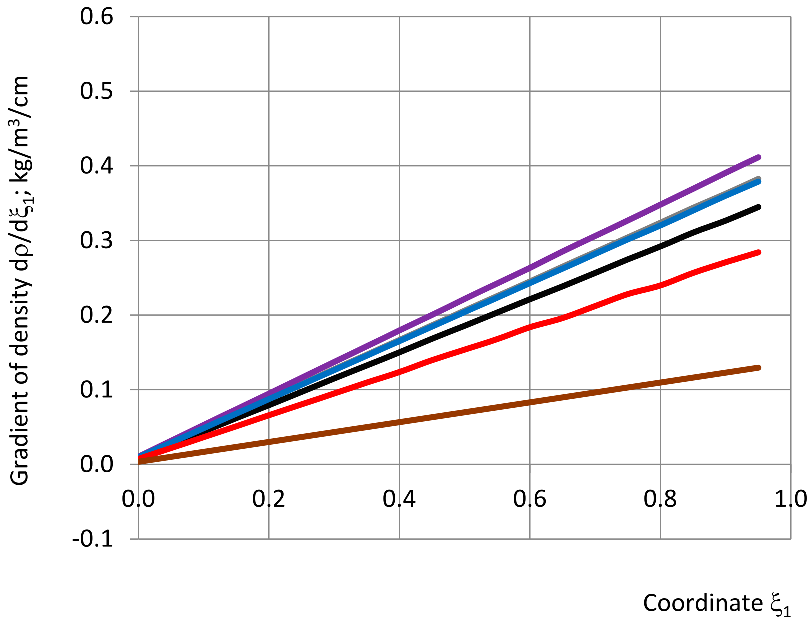

Figure S8. Density gradient in the vertical perpendicular section depending on coordinate ξ_1 at fixed values of coordinate ξ_3 1) $\xi_3 = 0.0$ (Black), 2) $\xi_3 = 0.22$ (Grey), 3) $\xi_3 = 0.44$ (Violet), 4) $\xi_3 = 0.67$ (Blue), 5) $\xi_3 = 0.89$ (Red), and 6) $\xi_3 = 1.00$ (Brown).

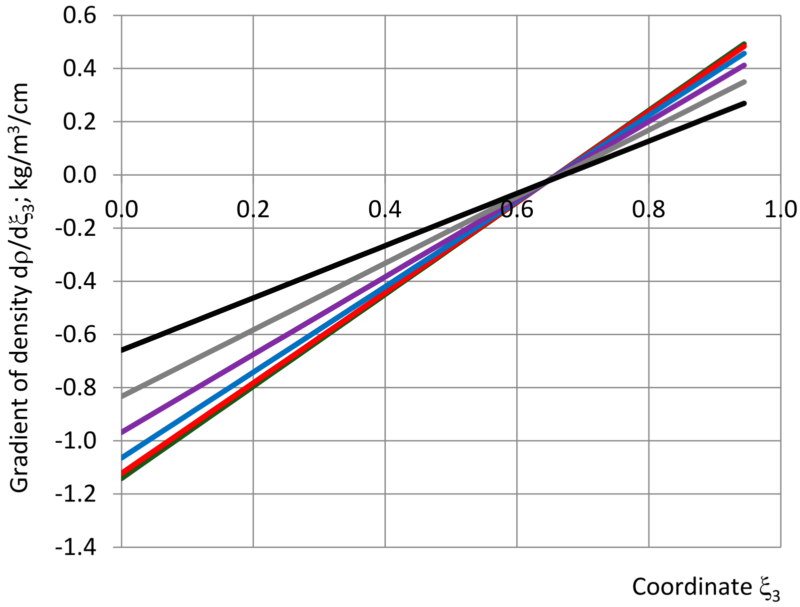

Figure S9. Density gradient in the vertical perpendicular section depending on coordinate ξ_3 at fixed values of coordinate ξ_1 1) ξ_1 = 0.0 (Black), 2) ξ_1 = 0.2 (Grey), 3) ξ_1 = 0.4 (Violet), 4) ξ_1 = 0.6 (Blue), 5) ξ_1 = 0.8 (Red), and 6) ξ_1 = 1.0 (Brown).

S10.Graphs of density and gradients in the vertical diagonal section

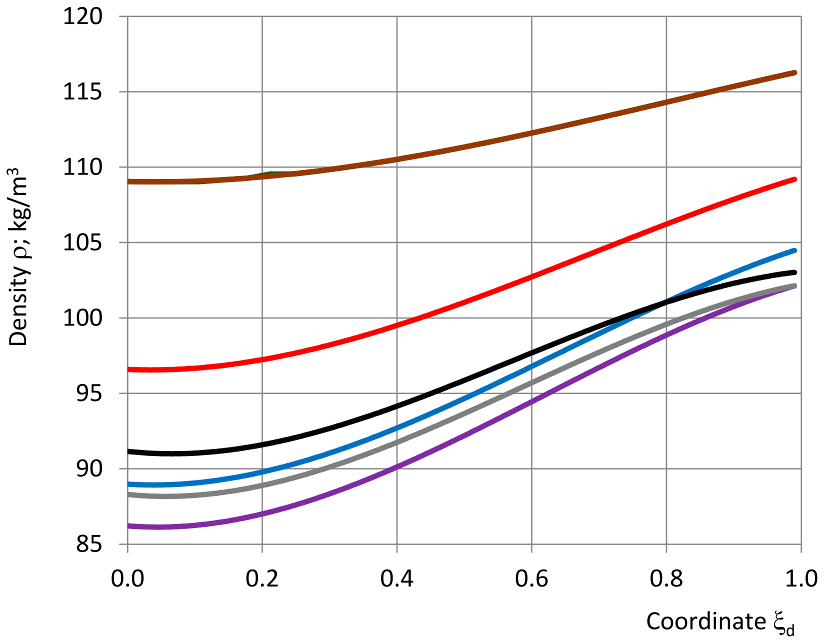

Figure S10. Density in the vertical diagonal section in dependence of coordinate ξ_d at fixed values of coordinate ξ_3 1) ξ_3 = 0.0 (Black), 2) ξ_3 = 0.22 (Grey), 3) ξ_3 = 0.44 (Violet), 4) ξ_3 = 0.67 (Blue), 5) ξ_3 = 0.89 (Red), and 6) ξ_3 = 1.00 (Brown).

https://doi.org/10.3390/polym18060733

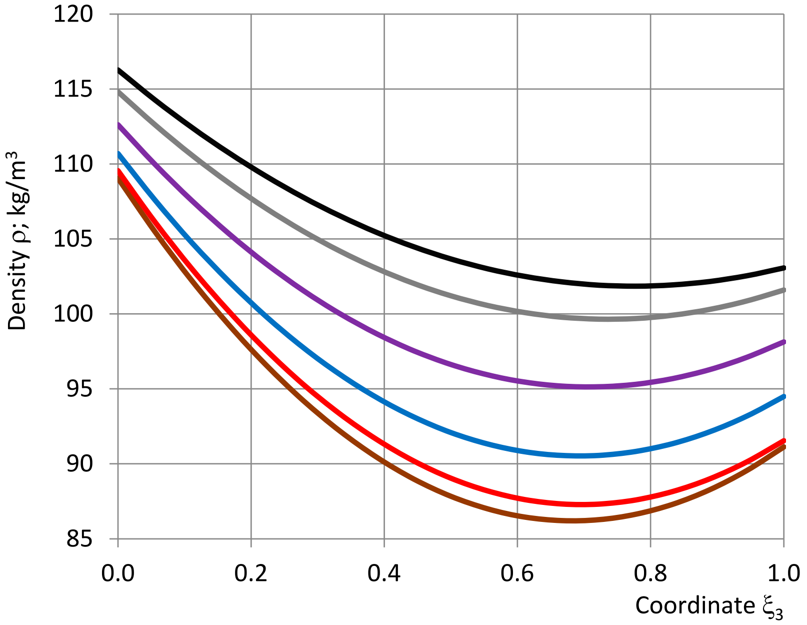

Figure S11. Density in the vertical diagonal section-in dependence of coordinate ξ_3 at fixed values of coordinate ξ_d 1) $\xi_d = 0.0$ (Black), 2) $\xi_d = 0.21$ (Grey), 3) $\xi_d = 0.42$ (Violet), 4) $\xi_d = 0.64$ (Blue), 5) $\xi_d = 0.85$ (Red), and 6) $\xi_d = 1.00$ (Brown).

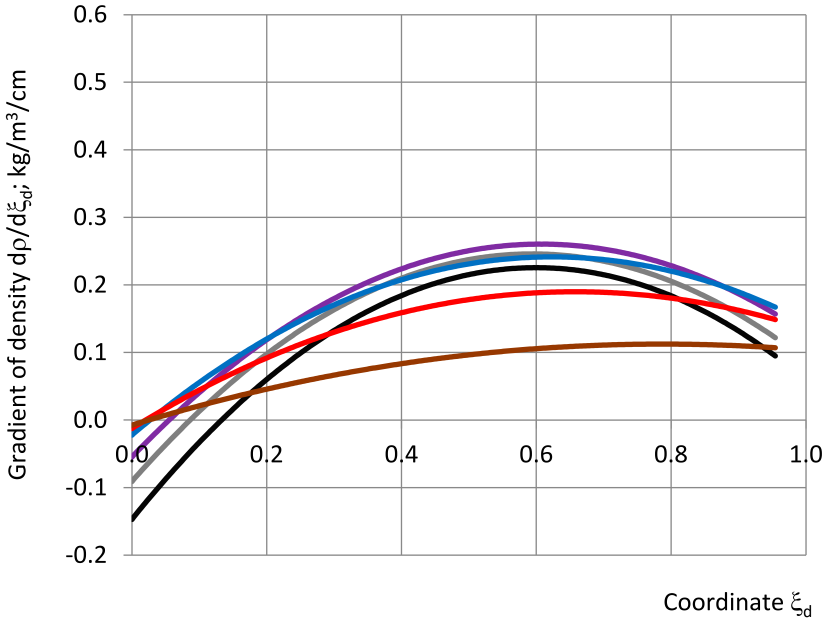

Figure S12. Density gradient in the vertical diagonal section depending on coordinate ξ_d at fixed values of coordinate ξ_3 1) $\xi_3 = 0.0$ (Black), 2) $\xi_3 = 0.22$ (Grey), 3) $\xi_3 = 0.44$ (Violet), 4) $\xi_3 = 0.67$ (Blue), 5) $\xi_3 = 0.89$ (Red), and 6) $\xi_3 = 1.00$ (Brown).

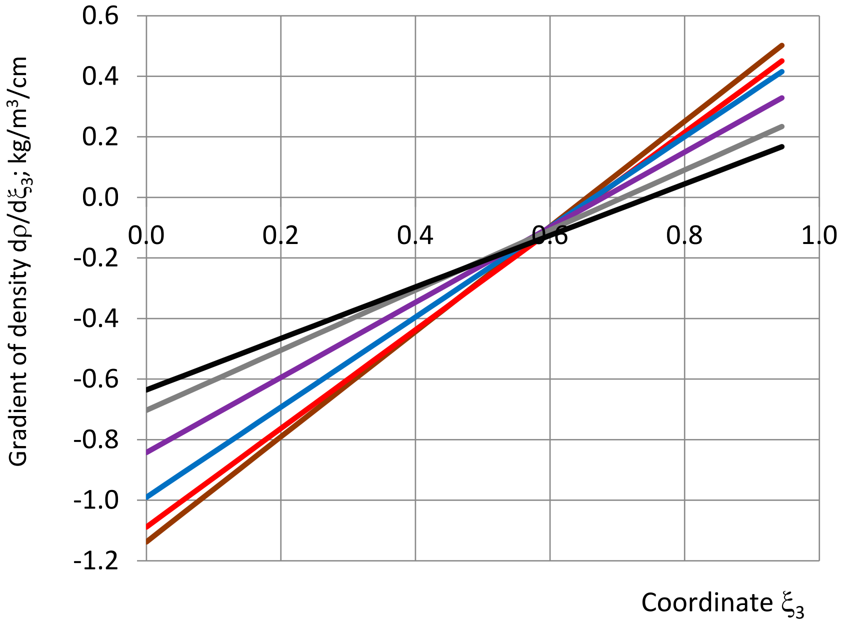

Figure S13. Density gradient in the vertical diagonal section depending on coordinate ξ_3 at fixed values of coordinate ξ_d 1) $\xi_d = 0.0$ (Black), 2) $\xi_d = 0.2$ (Grey), 3) $\xi_d = 0.4$ (Violet), 4) $\xi_d = 0.6$ (Blue), 5) $\xi_d = 0.8$ (Red), and 6) $\xi_d = 1.00$ (Brown).

https://doi.org/10.3390/polym18060733
